# Cerebrospinal fluid metabolomic signatures in paediatric MOGAD and POMS

**DOI:** 10.3389/fimmu.2025.1650785

**Published:** 2026-01-02

**Authors:** Piera De Gaspari, Stefano Sartori, Margherita Nosadini, Anna Sartori, Sara Mariotto, Roberto Assandri, Chiara Trabatti, Paola Cogo, Manuela Simonato

**Affiliations:** 1 Neuroimmunology group, Paediatric Research Institute “IRP Città della Speranza”, Padova, Italy; 2 Euroimmun Italy Laboratory, Euroimmun Italy, Padova, Italy; 3 Paediatric Neurology and Neurophysiology Unit, Department of Women’s and Children’s Health, University Hospital of Padova, Padova, Italy; 4 Department of Women’s and Children’s Health, University of Padova, Padova, Italy; 5 Neurology Unit, Department of Neurosciences, Biomedicine and Movement Sciences, University of Verona, Verona, Italy; 6 Clinical Investigation Laboratory, Maggiore Hospital, Azienda Socio Sanitaria Territoriale (ASST) Crema, Crema, Italy; 7 Pediatric and Neonatology Unit, Maggiore Hospital, Azienda Socio Sanitaria Territoriale (ASST) Crema, Crema, Italy; 8 PCare Laboratory, Pediatric Research Institute ‘Citta’ della Speranza’, Padova, Italy; 9 Department of Medicine, Division of Pediatrics, S. Maria della Misericordia University Hospital, University of Udine, Udine, Italy; 10 Department of Medicine-DIMED, University of Padova, Padova, Italy

**Keywords:** biomarkers, metabolites, MOGAD, neuroimmunology, POMS

## Abstract

**Introduction:**

Myelin oligodendrocyte glycoprotein (MOG) antibody-associated disease (MOGAD) and paediatric-onset multiple sclerosis (POMS) are acquired demyelinating syndromes (ADSs) that are increasingly recognised in paediatric care. They share clinical and magnetic resonance imaging (MRI) features, but they differ in prognosis and management, as POMS is a chronic inflammatory neurodegenerative disorder, whereas MOGAD is not. Early POMS diagnosis is essential to limit disability accumulation. Thus, early identification of these syndromes is key to their adequate management and family counselling and improving the outcome. Cerebrospinal fluid (CSF) biochemistry and cell count, oligoclonal band, IgG index, and serum MOG antibodies, together with brain and spine MRI, are the most valuable diagnostic biomarkers for differential diagnosis. However, it is not always possible to rely on these specific biomarkers to correctly identify these syndromes, especially at disease onset. In this perspective, metabolomic and lipidomic analyses have recently gained ground as a novel diagnostic approach.

**Methods:**

In the present study, high-sensitivity shotgun mass spectrometry was used to characterise the CSF metabolome and lipidome of children with MOGAD and POMS disorders compared with the CSF of children with non-demyelinating diseases used as controls.

**Results:**

The identification of 128 CSF hydrophilic metabolites and 210 lipids revealed characteristic changes in the relative metabolic concentrations in MOGAD compared with POMS, mainly related to the energy metabolism pathways. The lipidomic profile revealed the accumulation of the plasmalogens phosphatidylethanolamine (PE) and cholesterol esters as specific features of the lipid metabolic derangement. In this exploratory cohort, POMS showed higher very-long-chain PE and triglyceride (TG) signal intensities after false discovery rate (FDR) correction and effect size evaluation; however, these trends require confirmation in larger, independent cohorts.

**Conclusion:**

By exploring the CSF metabolomic profile, we demonstrated the usefulness of broad-range omic analysis as a fast and reliable method of biomarker discovery in children with demyelinating neurological disorders at the onset of the disease, which may be a valuable diagnostic complement to the existing biomarkers.

## 1 Introduction

Myelin oligodendrocyte glycoprotein (MOG) antibody-associated disease (MOGAD) and paediatric-onset multiple sclerosis (POMS) are two distinct acquired demyelinating syndromes (ADSs) occurring in children that can show clinical and imaging features overlapping at onset.

MOGAD is particularly frequent during childhood, representing approximately one-third of all first paediatric central nervous system (CNS) demyelinating events. Only 30%–50% of MOGAD cases are reported to be multiphasic in children, whereas POMS is characterised by chronic multiphasic inflammation with high relapse rates, potentially impacting cognitive and physical development. The diagnosis of POMS is based on the McDonald criteria, which have been validated in paediatric patients but more cautiously applied in children less than 11 years of age (1, 2). Differently, the diagnostic criteria for MOGAD have been newly proposed based on clinical syndrome, MOG-IgG status, and the exclusion of alternative diagnoses such as POMS and neuromyelitis optica spectrum disorder (NMOSD) (3). The discovery of pathogenic serum and/or cerebrospinal fluid (CSF) autoantibodies, targeted against MOG protein, enables their use as a diagnostic marker for the paediatric MOGAD phenotypes (4).

However, for many patients with ADSs, differential diagnosis is arduous in view of the possible clinical–radiological overlap at onset, and the introduction of therapy can be delayed (5). Thus, the lack of a single predictive or diagnostic test in these conditions remains a major obstacle in the patient’s care. Indeed, early differentiation between MOGAD and POMS is crucial due to significant prognostic and management differences, as POMS diagnosis allows early initiation of MS-specific preventive disease-modifying therapies (DMTs), while these may be ineffective or harmful in paediatric MOGAD (6–8). In recent years, there have been advances in molecular biology, cellular immunology, and omic technologies (genomics, proteomics, and metabolomics), which focus on exploring the processes underlying disease pathogenesis, to provide a set of possible biomarkers able to more precisely identify the ADS phenotype (9–11).

Metabolomics concerns the identification and quantification of small endogenous molecules in a biological system. Because metabolites represent substrates and final products of physiological processes in a living organism, the profiling of the metabolome in tissues and biofluids offers an instantaneous molecular image of the phenotype. Serum and CSF hydrophilic and hydrophobic compounds, analysed using high-resolution mass spectrometry (HRMS) techniques in the autoimmune process of ADSs, have been examined in adult patients, but the role of lipids, especially in combination with amino acids and short-chain organic acids, remains poorly defined (12). Lipids play a dual role in ADSs, both as substrates of myelin and as mediators of inflammation. Limited data are available on paediatric ADSs.

The focus of this study was to identify possible biomarkers able to specifically differentiate the major autoimmune disorders in children, such as MOGAD and POMS. With this aim, we investigated hydrophilic and hydrophobic metabolic profiles in CSF of children with these conditions at the disease onset, before any treatment was started, and we compared them to those obtained from the CSF of a pathological control group. We hypothesised that disturbances in CSF metabolite profiles reflected the myelin degradation/regeneration during the inflammatory process of brain tissue and that the mechanism could be different according to the disease process.

## 2 Materials and methods

### 2.1 Patients’ enrolment and sample collection

We included retrospective patients diagnosed with MOGAD (3) or POMS (1, 2) between 2006 and 2021 in Padova, Verona, or Crema hospitals, and with available onset serum and CSF samples (pre-treatment). As the control group, we selected children who underwent a lumbar puncture and blood tests for suspected increased intracranial hypertension and were finally diagnosed either with idiopathic intracranial hypertension (IIH) or headache. We excluded MOGAD and POMS patients who clinically did not fulfil Banwell (3) and Thompson (1) criteria, and for whom we did not have both serum and CSF samples available at the time of the diagnosis, from the study.

In total, 17 children affected by MOGAD (n = 8), POMS (n = 9), and IIH/pathological controls (n = 9) were selected. The patients were carefully characterised at both the clinical and laboratory levels. The serum and CSF samples were tested for all the neuronal and glial antibodies using in-house indirect immunohistochemistry, commercial indirect immunofluorescence, commercial cell-based assay (CBA), and commercially available line blot. All clinical and laboratory data were pooled and non-identifiable. The study was carried out according to the Declaration of Helsinki. The study was approved by the institutional review board and by the ethics committee of the Padova University Hospital 5046/AO/21.

### 2.2 Sample collection

All children underwent a lumbar puncture in the morning, after fasting for at least 6 hours. CSF samples were centrifuged immediately after collection (800 × *g* for 5 minutes) and aliquoted (100 µL per tube). All CSF samples were frozen at −80°C within 1 hour of lumbar puncture. Patient blood samples were allowed to clot at room temperature and then centrifuged at 3,500 × *g* for 10 minutes. Both CSF and serum samples were aliquoted within 1 hour of collection and stored at −80°C until analysis. Each aliquot underwent a single freeze–thaw cycle. For metabolomic analysis, CSF aliquots were thawed on ice and vortexed prior to processing. Sample collection and handling procedures were standardised and harmonised across all participating centres to ensure analytical consistency.

### 2.3 Sample characterisation

#### 2.3.1 Indirect immunohistochemistry and immunofluorescence

The presence of neuronal autoantibodies was checked by indirect immunohistochemistry using a commercial kit (FA 111m-3, Euroimmun AG, Luebeck, Germany) and following standard procedures (13). The results were evaluated using an optical microscope (Zeiss AxioPhot) at ×5, ×10, and ×20 magnification objectives. For the indirect immunofluorescence assay, commercial kits (FA 111m-3, FA 1111–1 Euroimmun AG) were used following the producer’s instructions. Slides were evaluated using a fluorescence microscope (Eurostar II, Euroimmun AG) at ×20 and ×40 magnification objectives.

#### 2.3.2 Line blot analysis

To confirm the presence of intracellular antibodies, a commercial line blot was used to test both the serum and CSF samples (DL1111–7 G, Euroimmun AG). The assay was performed in automation using EUROBlotOne and following the manufacturer’s instructions. The strips were then evaluated using the producer software (EUROLineScan). The cut-off was 15. Results under 15 were considered negative.

#### 2.3.3 CBA analysis

The confirmation of the presence of antigen surface antibodies was conducted using indirect immunofluorescence on cell lines transfected with vectors expressing the relevant antigens (CBA). Commercial CBAs were considered (FA112d-6 G; FA 1128 G, Euroimmun AG). Staining was performed following the producer’s instructions. The slides were then observed using a fluorescence microscope (Eurostar II, Euroimmun AG) at ×20 and ×40 magnification objectives.

### 2.4 Metabolomic and lipidomic analyses

#### 2.4.1 Sample preparation for HRMS

##### 2.4.1.1 Chemicals and reagents

Optima^®^ LC/MS grade water, methanol (MeOH), acetonitrile, 2-propanol, chloroform (CHCl_3_), and formic acid were from Sigma-Aldrich (St. Louis, MO, USA).

##### 2.4.1.2 Sample processing

CSF samples were extracted following the original protocol of Folch (14). Briefly, 100 µL of CSF was extracted in glass tubes by the addition of 1,600 µL of CHCl_3_:MeOH (2:1) and vortexed for 1 hour at 4°C. Afterwards, 300 µL of water was added, vortexed for 10 minutes, and centrifuged at 1,000 × *g* for 10 minutes at 4°C. After that, the lower lipid-rich phase (organic phase) was transferred into a clean glass tube. To the upper phase (aqueous phase), 1,000 µL of CHCl_3_:MeOH:H_2_O (86/14/1; v/v/v) was added, vortexed for 10 minutes, and centrifuged at 1,000 × *g* for 10 minutes at 4°C. The organic phases were combined, dried under nitrogen, and reconstituted with 50 μL of 50:50 isopropanol/water (organic phase). One thousand microliters of the aqueous phase was dried in a vacuum centrifuge, resuspended with 100 μL 95:5 water:acetonitrile with 0.1% formic acid, vortexed, sonicated for 60 seconds, and centrifuged at 13,000 × *g* for 20 minutes at 4°C.

Quality control (QC) samples were prepared by mixing equal volumes of all extracted samples. A procedural blank was used to monitor contamination acquired during all stages of sample preparation; a pool of the POMS, MOGAD, and control samples was also prepared.

#### 2.4.2 HRMS analysis

Untargeted metabolomic/lipidomic analysis was performed in CSF samples as reported in the **Supplementary Material**.

#### 2.4.3 Metabolomics workflow

A blank, consisting of the resuspension solvent, was included at the beginning and at the end of the run to test any possible contamination or carry-over effect. A quality control sample was injected every 10 injections throughout the run to monitor the sensitivity and stability of the platform. The procedural blank and the QC sample were used to condition the system at the beginning of the analysis. The order of injection of the samples was randomised to minimise the effect of the instrumental drift arising from column degradation or the contamination of the mass spectrometry source. All samples were analysed in full scan mode separately in positive and negative electrospray ionisation (ESI+/−). Data-dependent acquisition was performed on the pool of POMS, MOGAD, and control samples over three mass ranges (250–500, 501–700, and 701–1,200 m/z).

### 2.5 Data analysis

Raw data files were processed using the Compound Discoverer™ 3.3.2 software for initial data processing, including peak detection, peak alignment, and peak integration as reported by Simonato et al. (15) Raw files were aligned with an adaptive curve setting with 5-ppm mass tolerance and 0.4-min retention time shift. Unknown compounds were detected with a 5-ppm mass tolerance, 3 signal-to-noise ratio, and 30% relative intensity tolerance for isotope search and then grouped with 5-ppm mass and 0.2-min retention time tolerances. A procedural blank sample was used for background subtraction and noise removal during the pre-processing step. Peaks with less than a threefold increase, compared to blank samples, and those detected in less than 50% of QCs, and where the relative standard deviation (RSD) of the QCs was greater than 30% and the maximum QC corrected area relative standard deviation was greater than 25%, were removed from the list. QC-based normalisation was used to correct for intensity drift. A regression model was built based on the intensity drift of each metabolite in the QC samples and was used to predict and correct peak intensities of the same metabolite in subject samples. Metabolites identified in the processed raw data of mass spectral peaks were searched against both ChemSpider™ chemical structure database (3-ppm mass tolerance) and mzCloud and mzVault spectral library (precursor and fragment mass tolerance, 10 ppm). Five data sources were selected using the ChemSpider database: Human Metabolome Database (HMDB), Kyoto Encyclopedia of Genes and Genomes (KEGG), LIPID MAPS, BioCyc, and DrugBank. Lipid Search™ 5 was used to confirm lipid identity.

The Chemical Analysis Working Group defined four different levels of metabolite identification observed in the scientific literature. These included identified metabolites (level 1), putatively annotated compounds (level 2), putatively characterised compound classes (level 3), and unknown compounds (level 4). Our discriminating metabolites are all identified using both the molecular formula and the fragmentation data (level 2).

#### 2.5.1 Statistical analysis

Clinical patient data were summarised using frequency and percentage for binary variables, whereas mean and standard deviation or median and range (25th–75th) were presented for continuous numeric variables.

Alterations in each compound were evaluated using both Student’s t-test and the Mann–Whitney test. The “q-value” refers to the p-value adjusted to control the false discovery rate (FDR) using the Benjamini–Hochberg procedure. For each compound, group area fold change was calculated between groups and expressed on a log2 scale (Compound Discover™ 3.3).

## 3 Results

### 3.1 Clinical characteristics of the paediatric population

In this study, we considered 26 children to find possible biomarkers able to specifically identify and distinguish between MOGAD and POMS diagnoses. Considering the total number, we enrolled 15 girls and 11 boys, with a mean age at onset of 10 years (range 2–18 years). Of the patients affected by a demyelinating disease, eight had the diagnosis of MOGAD (eight girls and two boys), and nine had POMS (five girls and four boys). As a pathological control group, we considered nine patients (four girls and five boys), having the same mean age as the MOGAD and POMS group (range 4–14 years), affected by either IIH or headache. All the patients’ data at the disease onset are summarised in **Table 1**. Briefly, MOGAD patients presented acute demyelinating events such as acute disseminated encephalomyelitis (ADEM), optic neuritis, or myelitis, often accompanied by encephalopathy (four of eight cases) or motor deficits (four of eight cases). MRI findings were variable, with frequent supratentorial lesions and occasional optic nerve or spinal involvement. CSF oligoclonal bands were present in two of eight cases, and one of them had an Expanded Disability Status Scale (EDSS) score of 2 at follow-up. Generally, the clinical course included a few relapses with relatively low disability scores. POMS cases had clinically isolated syndromes, optic neuritis, or ADEM-like episodes. MRI frequently showed characteristic inflammatory lesions, and CSF commonly revealed intrathecal IgG synthesis (six of nine cases). These patients tended to have a more relapsing course than MOGAD, with some accumulating mild disability over time. IIH patients did not present any neurological symptoms at onset (i.e., seizures, encephalopathy, and motor deficits). Brain MRI findings were normal overall, and CSF analysis showed normal composition, albeit elevated intracranial pressure. The condition followed a benign, non-relapsing course, with no significant disability.

**Table 1:** General and clinical characteristics of the paediatric patients enrolled.

Diagnosis	Sex	Age at onset (years)	Clinical phenotype at first event	Seizures at first event	Encephalopathy at first event	Motor deficits at first event	EDSS score at first event	Brain MRI: supratentorial lesions	Brain MRI: infratentorial lesions	Brain MRI: contrast enhancement	Optic nerve MRI	Spine MRI	Days between disease onset and LP	Treatments received within 3 days prior to LP	CSF white blood cells/μL	CSF oligoclonal bands	Intracranial pressure (cmH_2_O)	Length of follow-up from onset (months)	Total number of events	EDSS at last follow-up
MOGAD	F	5.5	Encephalitis	Yes	No	No	3	Yes	No	No	Normal	Normal	5	Levetiracetam, electrolyte solution	25	Negative	n.a.	54	1	0
MOGAD	M	14.3	ADEM + ON	No	Yes	Yes	4.5	Yes	Yes	Yes	Abnormal	Abnormal	6	Electrolyte solution, pantoprazole, methylprednisolone (1 day prior to LP)	31	Intrathecal synthesis	n.a.	56	1	2
MOGAD	F	6.3	ON + myelitis	No	No	Yes	4.5	Yes	No	No	Abnormal	Abnormal	10	Electrolyte solution, ranitidine	2	Intrathecal synthesis	n.a.	78	3	0
MOGAD	F	15	ON	No	No	No	4	No	No	No	Abnormal	Normal	n.a.	None	Negative	Negative	Normal	1.5	1	2
MOGAD	F	18	ON	No	No	No	4	No	No		Abnormal	Normal	n.a.	n.a.	Negative	n.a.	n.a.	n.a.	2	n.a.
MOGAD	M	6	ADEM	No	Yes	Yes	n.a.	Yes	Yes		Normal	Abnormal	n.a.	n.a.	193	Negative	n.a.	2	1	0
MOGAD	F	2	ADEM	No	Yes	Yes	n.a.	No	Yes		Normal	Abnormal	n.a.	n.a.	5	n.a.	n.a.	8	1	0
MOGAD	F	9.5	ADEM	No	Yes	No	5	Yes	Yes	No	Normal	n.a.	n.a.	n.a.	26	n.a.	n.a.	24	1	0
POMS	F	13.2	CIS	No	No	No	1	Yes	No	Yes	n.a.	Abnormal	n.a.	n.a.	16	Intrathecal synthesis	n.a.	145	n.a.	n.a.
POMS	M	12.9	CIS	No	No	Yes	6	Yes	Yes	Yes	Normal	Abnormal	n.a.	n.a.	24	Intrathecal synthesis	n.a.	n.a.	n.a.	n.a.
POMS	F	3.6	ADEM	No	Yes	No	n.a.	Yes	Yes	Yes	Normal	Abnormal	n.a.	n.a.	8	Negative	n.a.	29	6	2
POMS	M	13.6	n.a.	n.a.	n.a.	n.a.	n.a.	Yes	No	Yes	Normal	n.a.	n.a.	n.a.	8	n.a.	n.a.	183	n.a.	n.a.
POMS	M	11.7	CIS	No	No	No	1	Yes	Yes	Yes	Normal	Normal	n.a.	n.a.	12	Intrathecal synthesis	n.a.	45	2	1
POMS	F	13.7	ON	No	No	No	No	Yes	Yes	No	Normal	Abnormal	n.a.	n.a.	2	Negative	n.a.	41	1	1
POMS	F	13.3	CIS	No	No	Yes	5.5	Yes	No	Yes	Normal	Normal	n.a.	n.a.	20	Intrathecal synthesis	n.a.	1	n.a.	n.a.
POMS	F	11	Psychotic event	No	No	No	2	Yes	No	n.a.	Normal	Normal	n.a.	Sertraline	9	Intrathecal synthesis	n.a.	23	2	1
POMS	M	11.4	CIS	No	No	Yes	6	Yes	No	Yes	Normal	Abnormal	10	n.a.	5	Intrathecal synthesis	n.a.	13	2	1
IIH	M	13.3	IIH	No	No	No	0	No	No	No	Normal	n.a.	4	Paracetamol	1	Negative	31	16	2	0
IIH	M	13.8	IIH	No	No	No	2	No	No	No	Normal	n.a.	n.a.	Electrolyte solution	4	Intrathecal synthesis	n.a.	18	1	0
IIH	F	12.9	IIH	No	No	No	2	No	No	No	Normal	n.a.	33	Acetazolamide, electrolyte solution	2	Negative	28	4	2	1
IIH	M	8.9	IIH	No	No	No	0	No	No	No	Normal	n.a.	7	Aripiprazole, valproate, lithium, melatonin, electrolyte solution	1	Negative	34	17	2	0
IIH	M	4.1	IIH	No	No	No	0	No	No	No	Normal	n.a.	3	Electrolyte solution	n.a.	n.a.	23	35	1	0
Headache	F	14.1	Headache	No	No	No	0	No	No	No	Normal	n.a.	15	Electrolyte solution	2	Negative	25	12	1	0
IIH	F	10.9	IIH	No	No	No	0	No	No	No	Normal	Normal	n.a.	Electrolyte solution	3	Negative	45	23	n.a	0
IIH	F	10.8	IIH	No	No	No	1	No	No	No	Normal	Normal	22	Electrolyte solution	0	Negative	58	15	2	0
IIH	M	11.8	IIH	No	No	No	2	No	No	No	Normal	n.a.	n.a.	Electrolyte solution, ondansetron, paracetamol, melatonin	0	Negative	28	8	1	1

The table summarises the final diagnosis, sex, age, clinical syndrome, and total number of events of the paediatric patients enrolled in our study. The age is expressed in years. The clinical syndrome refers to the onset of the disease. The total number of events includes the onset of the disease.

MOGAD, myelin oligodendrocyte glycoprotein antibody-associated disease; ADEM, acute disseminated encephalomyelitis; ON, optic neuritis; POMS, paediatric-onset multiple sclerosis; CIS, clinically isolated syndrome; N/A, not available; IIH, idiopathic intracranial hypertension; EDSS, Expanded Disability Status Scale; CSF, cerebrospinal fluid; LP, lumbar puncture.

From all the patients, both serum and CSF paired “naïve” samples, meaning biological specimens obtained before treatment, were available.

#### 3.1.1 Sample pre-characterisation

**Figure 1:**
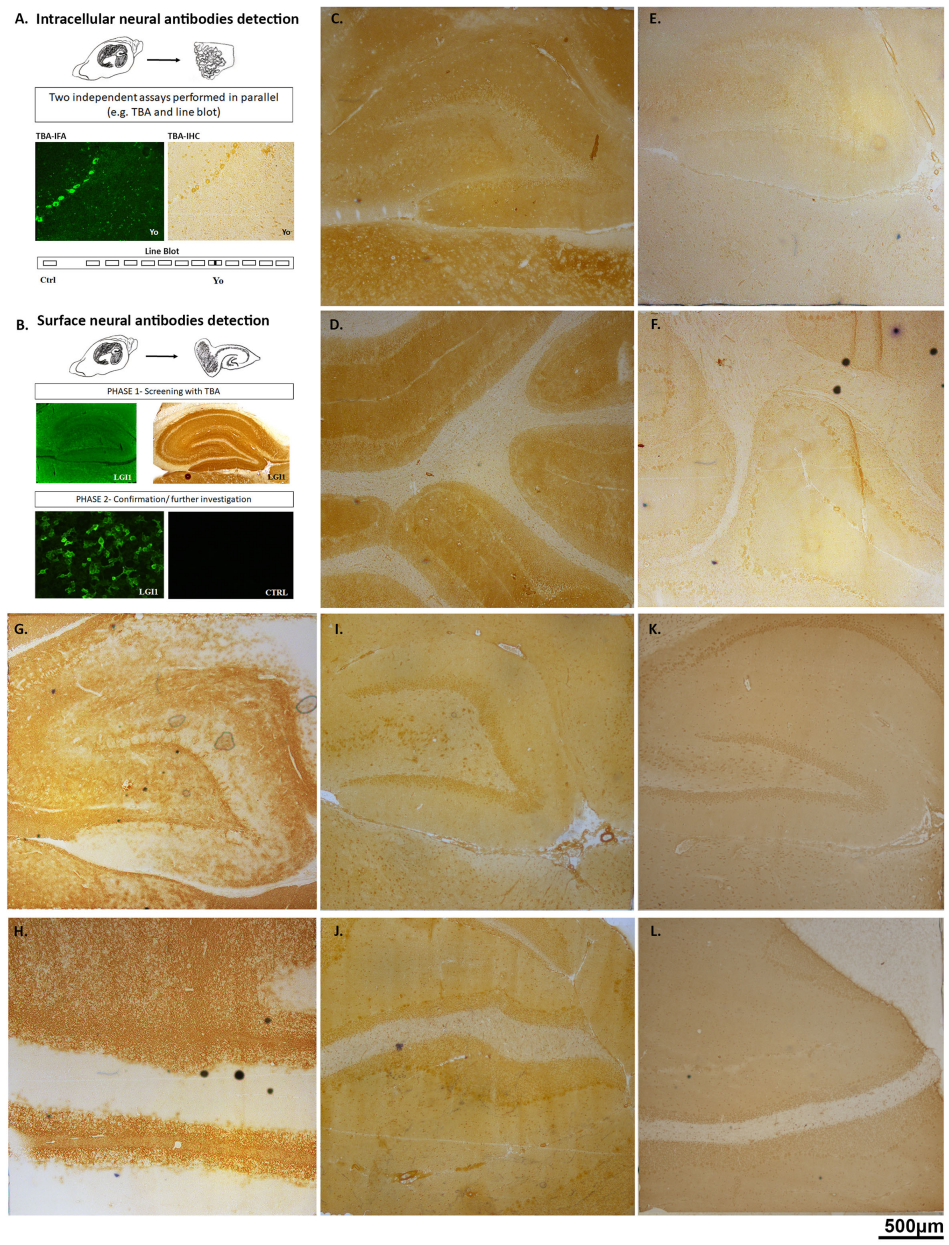
Indirect immunohistochemistry analysis. Schematic representation of the diagnostic laboratory approach performed to detect neuronal antibodies **(A, B)**. Representative rat brain and cerebellum images with ×5 magnification of a positive control sample **(C, D)**, a negative control sample **(E, F)**, a MOG sample **(G, H)**, a POMS sample brain **(I, J)**, and an IIH sample **(K, L)**. MOG, myelin oligodendrocyte glycoprotein; POMS, paediatric-onset multiple sclerosis; IIH, idiopathic intracranial hypertension.

**Figure 2:**
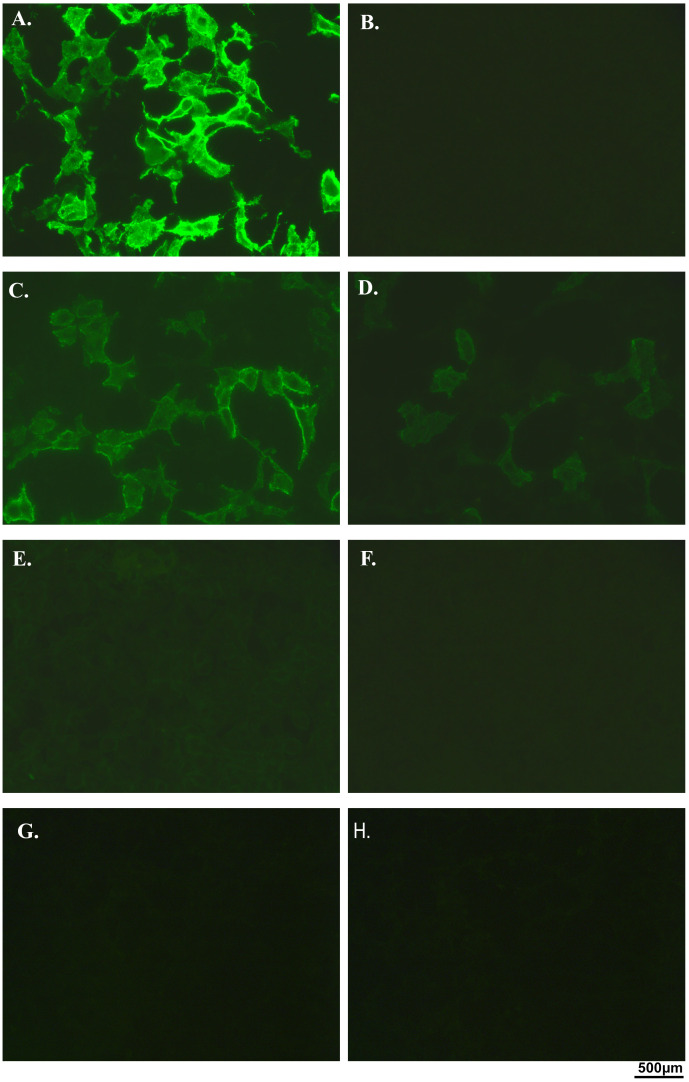
Cell-based assay (CBA) analysis of serum and CSF paediatric samples. The presence of MOG antibodies has been investigated using a commercial cell-based assay. Representative images of serum and CSF samples of control patients negative for NMDAR CBA **(A, B)**. Representative images of positive and negative controls **(A, B)**. Serum and CSF of a MOGAD patient, both positive for the presence of MOG antibodies **(C, D)**. Serum and CSF of a POMS patient **(E, F)** and of an IIH patient **(G, H)**, all negative for MOG antibodies. All the images were taken with a ×40 magnification objective. CSF, cerebrospinal fluid; MOG, myelin oligodendrocyte glycoprotein; MOGAD, MOG antibody-associated disease; POMS, paediatric-onset multiple sclerosis; IIH, idiopathic intracranial hypertension; NMDAR, N-methyl-D-aspartate receptor.

The diagnosis of MOGAD, POMS, and IIH is based on the clinical features; however, the detection or absence of neuronal antibodies is an additional tool that could support the definite diagnosis when the clinical presentation is not so clear or in case of overlap syndrome. Thus, all the serum and CSF samples of the 26 patients were first screened for the general presence of antineuronal antibodies using indirect immunohistochemistry (IHC) and immunofluorescence assay (IFA). In IHC, samples of MOGAD patients showed an intense myelin staining in the granular layer and the white matter of the cerebellum and the rat brain. Serum samples showed a higher clear intensity compared to CSF ones. Multiple sclerosis samples showed a non-specific pattern highlighting neurofilaments and myelin. IIH samples were negative and did not show any staining in both the rat hippocampus and rat cerebellum (**Figure 1**). In our cohort, IFA mirrored the same patterns observed via IHC analysis (**Supplementary Figure 1**). The results obtained suggested that our MOGAD patients’ samples indeed presented surface glial–neuronal antibodies. However, it was known that in IFA, a high auto-fluorescence background was present, and the interpretation of the presence or absence of a specific staining may be more difficult, requiring higher expertise. In addition, neither of these techniques is the first approach to be considered for the detection of MOG antibodies, as the conformational nature of the antigenic epitope means that MOG antibodies cannot always be recognised using this method (16). Therefore, a specific commercial cell-based assay was also performed, and MOG-IgG was detected in all MOGAD serum and CSF samples and not detected in POMS or IIH; assay performance characteristics in this cohort were not established (**Figure 2**). Furthermore, to possibly exclude the concurrent presence of other anti-neuronal antibodies, all the samples were also analysed using a line blot and a more comprehensive CBA panel assay (**Supplementary Figures 2a, b**). They all showed negative results. The presence of MOG antibodies only in MOGAD patients clearly supported the clinical phenotype of these patients, helping to distinguish them from the POMS and control groups. Samples belonging to patients with multiple sclerosis (MS) lacked a specific brain or cerebellum pattern, and they tested negative in CBA and blot. These results confirmed that, up to now, none of the known neuronal antibodies having intracellular and surface targets have been specifically linked to MS disease. In addition, the reference techniques normally used to identify neuronal antibodies seem not to be suitable for identifying specific biomarkers in MS samples.

#### 3.1.2 HRMS results

##### 3.1.2.1 Polar metabolites in CSF samples of MOGAD and POMS patients

**Figure 3:**
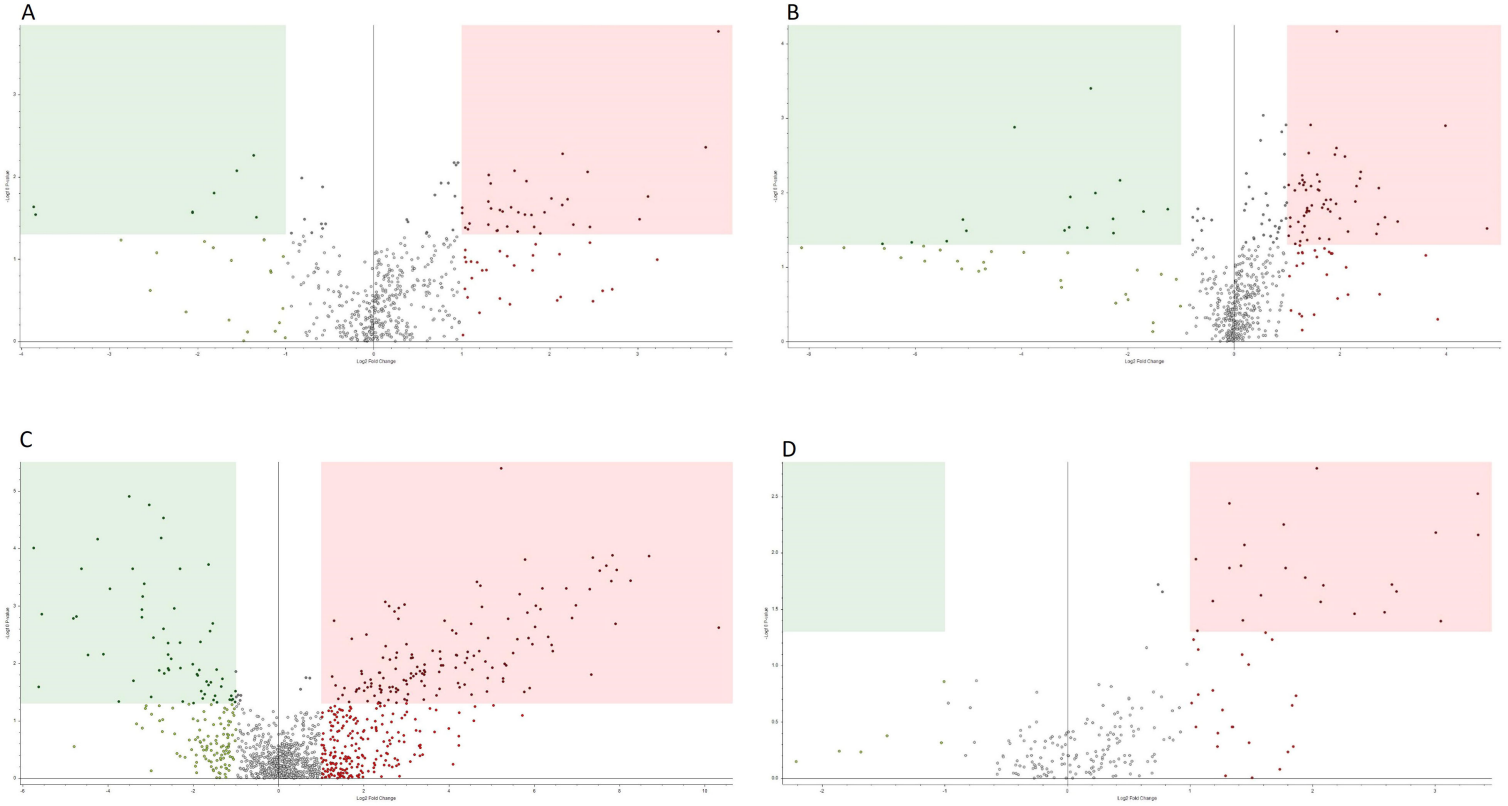
Volcano plots for the MOGAD vs. POMS samples. Volcano plot (ratio MOGAD/POMS) in positive (ESI+; **A, C**) and negative (ESI−; **B, D**) modes. Polar compounds are in the upper part of the figure, whereas non-polar compounds are in the lower. Fold change (log2) on X-axis plotted against p-value (−log10) on Y-axis. The horizontal line marks the p = 0.05, and the vertical lines mark a fold change of ±1.0. All plots in the red quadrant indicate significantly upregulated metabolites in the MOGAD samples with respect to POMS, whereas all plots in the green quadrant indicate the opposite. MOGAD, myelin oligodendrocyte glycoprotein antibody-associated disease; POMS, paediatric-onset multiple sclerosis; ESI, electrospray ionisation.

A total of 1,042 and 1,411 compounds with unique molecular weight and retention times were annotated from the positive and negative modes, respectively. After data cleaning to remove unreproducible features (>30% RSD of QC) and background subtraction, 531 (ESI+) and 591 (ESI−) compounds remained. Of these, 44 and 75 compounds had a p-value <0.05 and a group area fold change (expressed as log2) <−1 or >1 (volcano plot, **Figures 3A, B**).

##### 3.1.2.2 Non-polar metabolites in CSF samples of MOGAD and POMS patients

From the MOGAD vs. POMS experiments, a total of 14,075 and 1,980 compounds with unique molecular weight and retention times were annotated from the positive and the negative modes, respectively. After data cleaning to remove unreproducible features (>30% RSD of QC) and background subtraction, 1,186 (ESI+) and 215 (ESI−) compounds remained (volcano plot, **Figure 3**). Of these 175, 23 compounds had a p-value <0.05 and a group area fold change (expressed as log2) <−1 or >1 (volcano plot, **Figures 3C, D**). In the **Supplementary Material**, the corresponding Sparse Partial Least Squares–Discriminant Analysis (sPLS-DA) plots are provided (**Supplementary Figure 3**) as well as the identification of differential CSF metabolites in MOGAD and POMS patients.

The differential compounds (119 polar and 198 non-polar) were filtered to retain only those with at least one associated fragmentation spectrum, resulting in the exclusion of 202 compounds. Subsequently, compounds lacking a tentative identification name (N = 63) were removed. The remaining 52 compounds were visually inspected to identify duplicates and those with poor peak quality. Ultimately, our analysis led to the identification of 28 metabolites in human CSF samples (**Table 2**). More information about chemical identifiers, group area, and identification level is detailed in **Supplementary Table 2**. The identification corresponded to levels 2 and 3 of the metabolomics standard initiative (17). Glutamate and capryloyl glycine were recognised in both polarities. Most of the metabolites were classified as amino acids and peptides (glutamic acid, isoleucine, 3-methylhistidine, glutamine, hexanoylglycine, and capryloyl glycine) and dicarboxylic acids (ethylmalonic acid, mesaconic acid, azelaic acid, and suberic acid).

**Table 2:** Putatively annotated metabolites.

Metabolite	Ion description	Detected m/z	Predicted formula	Δmass (ppm)	RT (min)	RSD QC (%)	p-Value	q-Value	Ratio (MOGAD/POMS)
PE (P-18:0_22:6)	[M+H]+	776.55	C_45_H_78_NO_7_P	0	11.809	16	0.0274	0.0457	69.358
Pipercitine	[M+H]+	349.33	C_23_H_43_NO	0.26	5.555	8	0.0152	0.0304	41.986
2,2′-(2,6-Pyridinediyl)bis(*N*,*N*,*N*′,*N*′-tetramethyl-1,3-propanediamine)	[M+H]+	336.31	C_19_H_37_N_5_	−0.62	5.023	22	0.0016	0.0117	25.167
(4*E*)-4-(Hexadecylimino)pentanoic acid	[M+H]+	340.32	C_21_H_41_NO_2_	0.28	4.891	11	0.036	0.0539	21.951
DG (P-6:0_17:0)	[M+H]+	427.38	C_26_H_50_O_4_	−0.03	6.302	24	0.0006	0.0086	20.932
CE 18:2	[M+Na]+	671.57	C_45_H_76_O_2_	−0.42	20.174	22	0.0207	0.0658	19.585
10-Hydroxydecanoic acid	[M−H]−	187.13	C_10_H_20_O_3_	0.93	0.717	4	0.0047		15.958
*N*,*N*-Dimethyl-l-histidine	[M+H]+	184.11	C_8_H_13_N_3_O_2_	0.46	2.585	2	<0.0001	0.0025	14.935
*N*-Methyl-*N*-(1-phenylethyl)-1-hexadecanamine	[M+H]+	360.36	C_25_H_45_N	−0.02	3.749	19	0.0464	0.0557	6.817
11-Aminoundecanoic acid	[M+H]+	202.18	C_11_H_23_NO_2_	0.32	3.151	1	0.0111	0.0256	5.322
d-Glutamate	[M+H]+	148.06	C_5_H_9_NO_4_	0.57	6.77	17	0.0111	0.0256	3.808
d-Glutamate	[M–H]−	146.05	C_5_H_9_NO_4_	1.05	6.747	2	0.0464	0.0557	4.177
Capryloyl glycine	[M–H]−	200.13	C_10_H_19_NO_3_	1.28	0.713	4	0.0274	0.0457	3.252
Isoleucine	[M+H]+	132.1	C_6_H_13_NO_2_	0.64	3.357	3	0.0111	0.0256	3.093
Ethylmalonic acid	[M–H]−	131.04	C_5_H_8_O_4_	0.49	2.976	14	0.0025	0.0123	2.702
Nicotinamide	[M+H]+	123.06	C_6_H_13_N_2_O	0.01	0.794	4	0.0206	0.0387	2.444
Suberic acid	[M–H]−	173.08	C_8_H_14_O_4_	0.84	0.741	4	0.0016	0.0117	2.437
Azelaic acid	[M–H]−	187.1	C_9_H_16_O_4_	1.19	0.708	5	0.0025	0.0123	2.415
Capryloyl glycine	[M+H]+	230.18	C_12_H_23_NO_3_	0.39	0.65	3	0.0079	0.0256	2.102
Piperidine	[M+H]+	86.096	C_5_H_11_N	0.1	3.087	2	0.0464	0.0557	2.087
*N*-Acetyl-1-aspartylglutamic acid	[M+H]+	305.1	C_11_H_16_N_2_O_8_	−0.27	6.093	2	0.0464	0.0557	2.046
PE 40:5	[M+H]+	794.57	C_45_H_80_NO_8_P	0.35	12.109	11	0.0342	0.0775	0.349
PE 40:4	[M+H]+	796.58	C_45_H_82_NO_8_P	−0.37	12.851	15	0.0079	0.0256	0.317
*N*^8^-Acetylspermidine	[M+H]+	188.18	C_9_H_21_N_3_O	0.72	7.668	5	0.065	0.0696	0.257
Mesaconic acid	[M–H]−	129.02	C_5_H_6_O_4_	1.1	5.251	5	0.0464	0.0557	0.21
TG (18:1_20:1_18:2)	[M+NH_4_]+	928.83	C_59_H_106_O_6_	−0.95	21.55	13	0.0111	0.0256	0.163
Citric acid	[M–H]−	191.02	C_6_H_8_O_7_	1.21	5.252	4	0.0152	0.0658	0.146
3-Methylhistidine	[M+H]+	170.09	C_7_H_11_N_3_O_2_	0.56	8.343	8	0.0152	0.0304	0.071
Theobromine	[M+H]+	181.07	C7H8N4O2	0.09	8.343	3	0.036	0.0539	0.069
Ascorbic acid	[M–H]−	175.02	C_6_H_8_O_6_	0.95	0.71	5	0.0464	0.5414	0.045

Summary of the most relevant putatively annotated metabolites analysed using high-definition mass spectrometry (HDMS) in electrospray ionisation (ESI) negative mode in cerebrospinal fluid (CSF) samples from patients with paediatric-onset multiple sclerosis (POMS) and myelin oligodendrocyte glycoprotein antibody-associated disease (MOGAD). Metabolite annotations correspond to identification level 2. p-Values were calculated using the Mann–Whitney test. The “q-value” is the p-value that has been adjusted for the false discovery rate using the Benjamini-Hochberg procedure. The ratio was obtained by dividing the metabolite area in the MOGAD group by that in the POMS group. A ratio greater than 1 indicates that the metabolite is more abundant in CSF samples from MOGAD patients compared to POMS, whereas a ratio lower than 1 indicates the opposite.

PE-P, alkenyl ether glycerolphosphoethanolamine; DG-P, alkenyl ether diacylglycerol; CE, cholesterol ester; TG, triglyceride; RSD, relative standard deviation; QC, quality control; RT, retention time; PE, phosphatidylethanolamine.

Among the 28 discriminant metabolites, 19 were higher in MOGAD patients with respect to POMS.

#### 3.1.3 Differential metabolites in controls versus patients

Since the CSF physiologic metabolic profile is currently unknown in children, as are the metabolic changes induced by MOGAD and POMS diseases, we analysed the CSF metabolic profiles of control patients to compare them with the differential metabolites found in MOGAD vs. POMS.

##### 3.1.3.1 Polar metabolites

**Figure 4:**
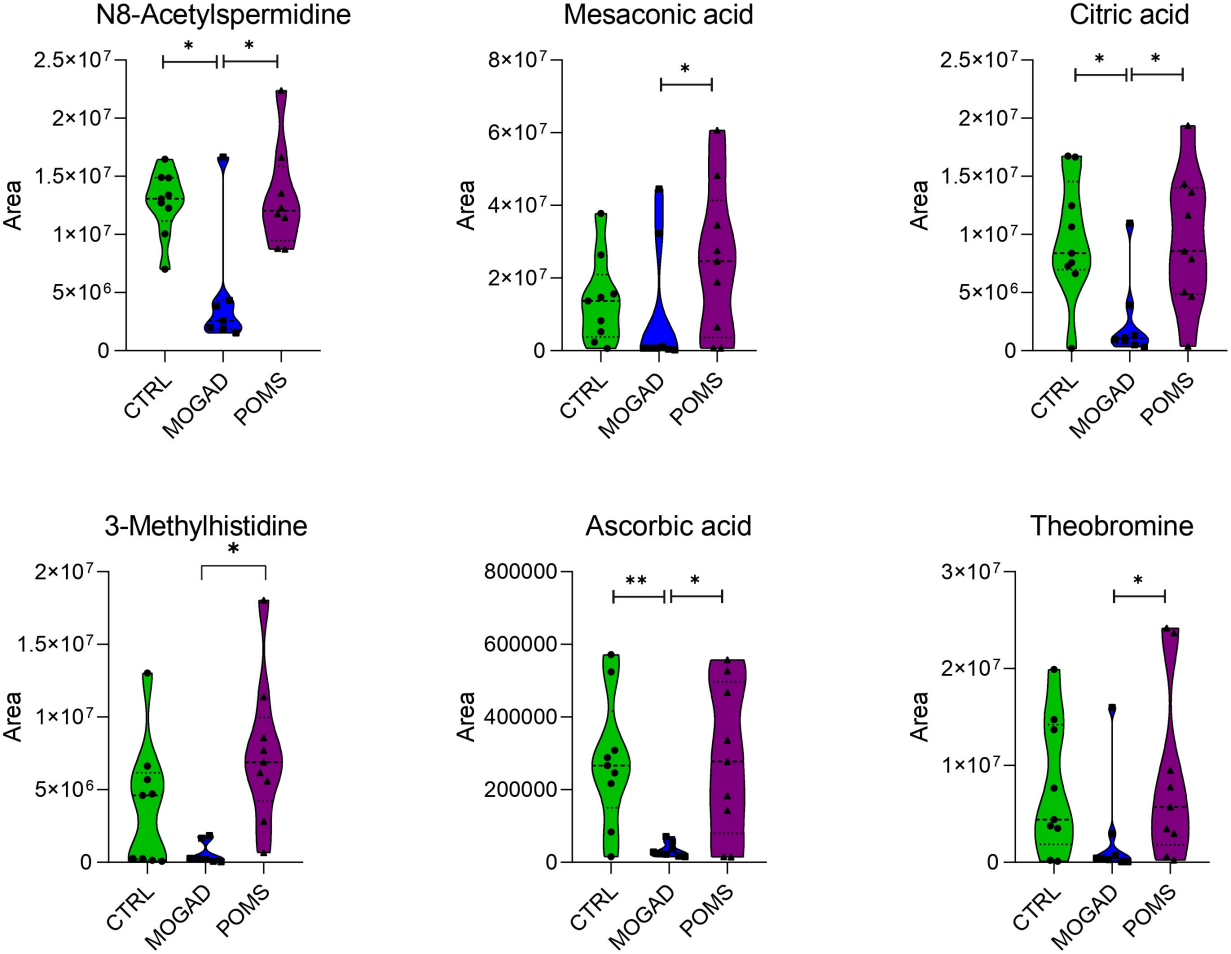
Polar metabolites significantly decreased in the MOGAD cohort. Violin plots showing the median (central dashed line) and the first and third quartiles (lower and upper dotted lines) of the most representative metabolites identified in cerebrospinal fluid (CSF) from control, MOGAD, and POMS patients. Comparisons were performed using the Mann–Whitney test (MOGAD vs. POMS, MOGAD vs. control, and POMS vs. control). Each point represents an individual sample. The plots illustrate the median, interquartile range, and overall data density. Statistical significance is indicated as follows: p < 0.05 (*), p < 0.01 (**), and p < 0.001 (***). MOGAD, myelin oligodendrocyte glycoprotein antibody-associated disease; POMS, paediatric-onset multiple sclerosis.

Within the polar metabolites, the median area of *N^8^*-acetylspermidine, mesaconic acid, citric acid, 3-methylhistidine, ascorbic acid, and theobromine was significantly lower in MOGAD patients with respect to both POMS and control patients (**Figure 4**).

**Figure 5:**
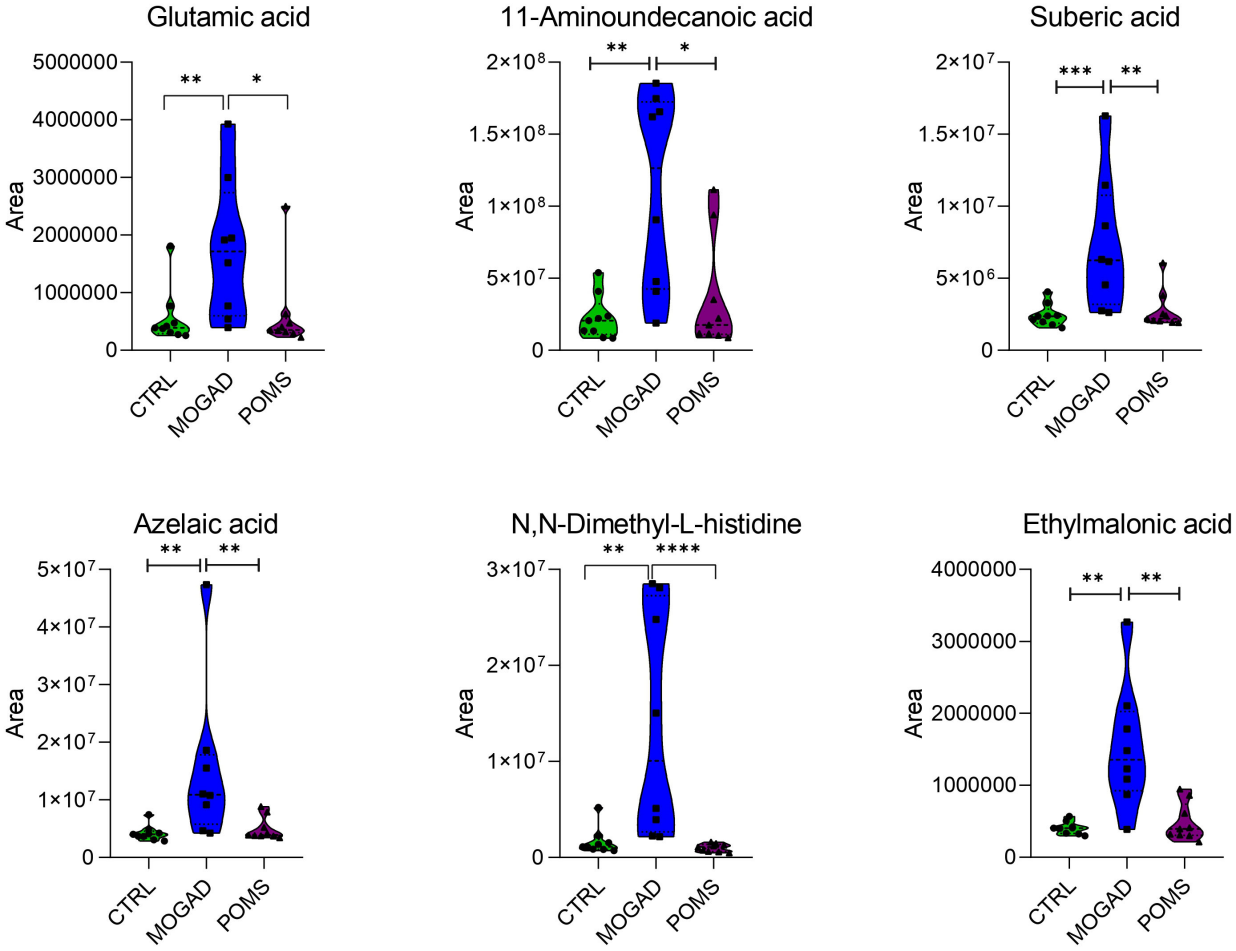
Polar metabolites significantly increased in the MOGAD cohort. Violin plots showing the median (central dashed line) and the first and third quartiles (lower and upper dotted lines) of the most representative metabolites identified in cerebrospinal fluid (CSF) from control, MOGAD, and POMS patients. Comparisons were performed using the Mann–Whitney test (MOGAD vs. POMS, MOGAD vs. control, and POMS vs. control). Each point represents an individual sample. The plots illustrate the median, interquartile range, and overall data density. Statistical significance is indicated as follows: p < 0.05 (*), p < 0.01 (**), and p < 0.001 (***). MOGAD, myelin oligodendrocyte glycoprotein antibody-associated disease; POMS, paediatric-onset multiple sclerosis.

The median area of the following polar metabolites was significantly higher in the MOGAD group with respect to both the POMS and control groups: l-glutamic acid, 11-aminoundecanoic acid, ethylmalonic acid, suberic acid, azelaic acid, isoleucine, nicotinamide, capryloyl glycine, piperidine, 10-hydroxyundecanoic acid, *N*,*N*-dimethyl-l-histidine, and *N*-acetyl-1-aspartylglutamic acid. The most representative are depicted in **Figure 5**.

##### 3.1.3.2 Non-polar (lipid metabolites)

**Figure 6:**
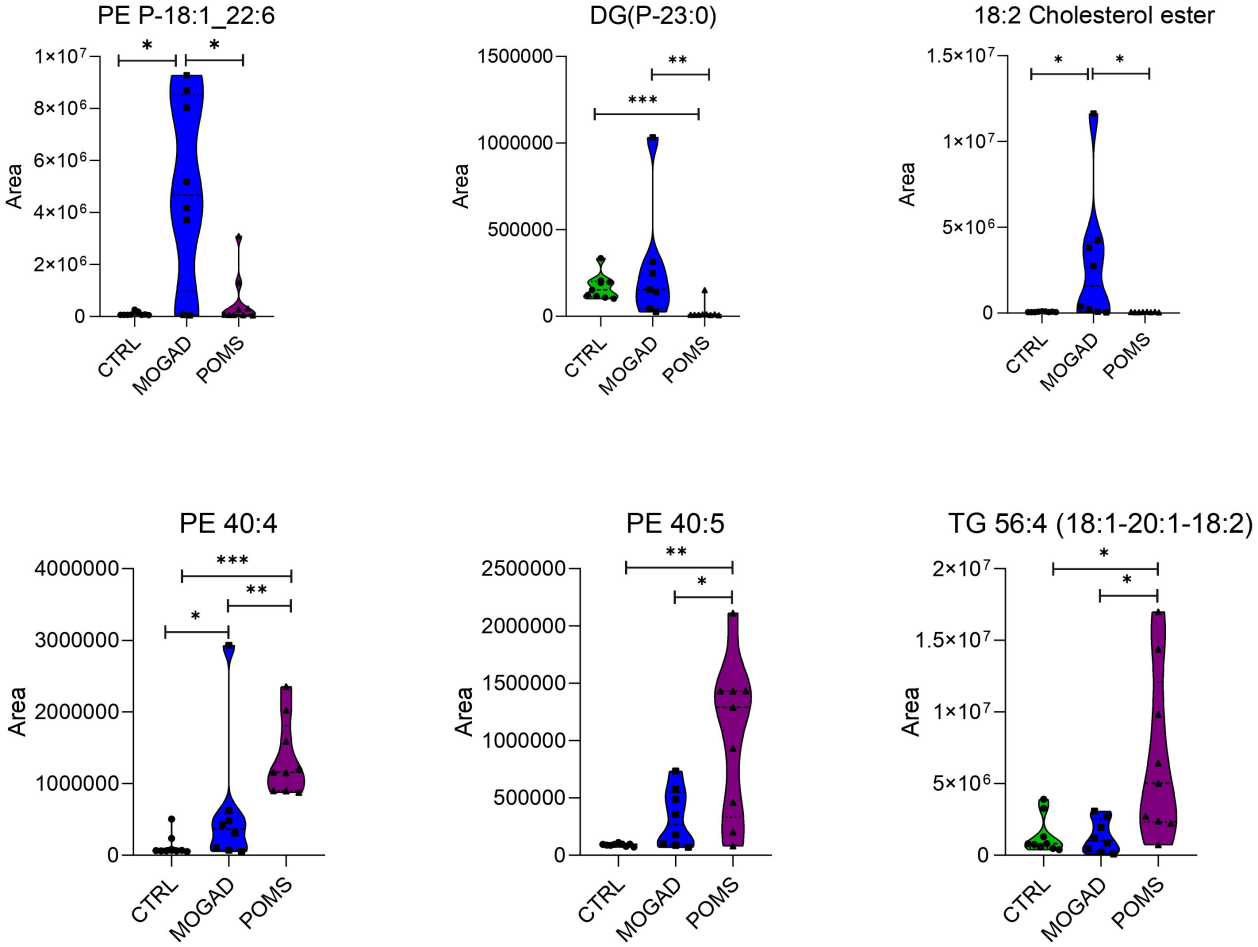
The most significant non-polar (lipid) metabolites in the MOGAD cohort. Violin plots showing the median (central dashed line) and the first and third quartiles (lower and upper dotted lines) of the most representative metabolites identified in cerebrospinal fluid (CSF) from control, MOGAD, and POMS patients. Comparisons were performed using the Mann–Whitney test (MOGAD vs. POMS, MOGAD vs. control, and POMS vs. control). Each point represents an individual sample. The plots illustrate the median, interquartile range, and overall data density. Statistical significance is indicated as follows: p < 0.05 (*), p < 0.01 (**), and p < 0.001 (***). PE, phosphatidylethanolamine; PE P, alkenyl ether glyceroPE; DG P, alkenyl ether diacylglycerol; TG, triglyceride; MOGAD, myelin oligodendrocyte glycoprotein antibody-associated disease; POMS, paediatric-onset multiple sclerosis.

Within the non-polar compounds, the median area of alkenyl ether glycerophosphatidylethanolamine (PE P)-18:0_22:6, alkenyl ether diacylglycerol (DG P)-23:0, and cholesterol ester (CE) (18:2) was higher in the MOGAD group with respect to both the POMS and control groups. In contrast, the median area of PE 40:4, PE 40:5, and triacylglycerol (TG) 56:4 was lower in the MOGAD group (**Figure 6**). The “P-” prefix was used for the 1*Z*-akenyl ether (plasmalogen) substituent.

## 4 Discussion

Our study primarily aimed to identify variances in metabolites within the CSF of children at the onset of MOGAD syndrome as compared to those of children at the onset of POMS and those with no brain autoimmune and inflammatory diseases. Additionally, we aimed to elucidate the underlying reasons for these alterations from a pathophysiological perspective. For this purpose, patients were strictly selected and carefully characterised at both the clinical and laboratory levels. Indeed, we wanted to investigate highly homogenous MOGAD and POMS patient groups and controls at the early stages of the disease, not having any other possible interfering factors (i.e., overlap syndromes and further neuronal antibodies). These variables may have affected our metabolomic results, leading to confounding and inaccurate results.

In this study, we have shown statistically significant changes in the metabolomics and lipidomics of CSF in MOGAD patients compared with POMS and control patients. The difference in metabolite levels we found in the two diseases sustains and reflects their distinct nature and pathological mechanisms behind their development.

### 4.1 Amino acid metabolism

In our study, we detected alterations in amino acid metabolism in both MOGAD and POMS. Key metabolites such as histidine and its derivatives demonstrated differential regulation across cohorts. In particular, 3-methylhistidine was higher in POMS and *N*,*N*-dimethyl-l-histidine in MOGAD.

Histidine is an essential amino acid and the precursor of histamine, a brain neurotransmitter, important for normal daily physiological functions. Histidine has a clear neuroprotective role; after an injury, it promotes astrocyte migration, ameliorating the glial scar area (18). Low levels of histidine have been associated with depression, psychomotor disability, increased tiredness, and impaired cognitive functions (19). In addition, Martynova et al. demonstrated that low levels of histidine have an inverse correlation with inflammation in MS patients, leading to the conclusion that histidine also has immune-modulator and anti-inflammatory properties (20, 21). The metabolite 3-methylhistidine is widely recognised as a marker of muscle turnover and myofibrillar protein degradation. Patients affected by neuromuscular disease, such as myasthenia gravis or Duchenne syndrome, have shown high levels of this metabolite (22). The hallmarks of MS are demyelination and neurodegeneration; however, recently, it has been suggested that the skeletal muscles are affected. People with MS can experience chronic mobility reduction and fatigue, leading to a sedentary lifestyle, causing loss of muscle mass, strength, and contractility (23). Thus, the high level we found in POMS can match the idea that, beyond the neuronal degeneration, muscle damage is also occurring. Differently, in MOGAD patients, muscle catabolism is not involved, and the prominent process is demyelination. Thus, we can speculate that in our MOGAD cohort, the high level of *N*,*N*-dimethyl-histidine may represent histidine’s immunomodulatory and neuroprotective activities, consistent with the acute inflammatory profile of this disorder.

In addition, our MOGAD cohort showed high levels of glutamic acid and low levels of ascorbic acid. Glutamic acid is the most important excitatory neurotransmitter in the mammalian brain. A connection between glutamatergic activity and ascorbic acid in the CNS has already been demonstrated (24). Mi and colleagues showed that ascorbate has a fundamental role in protecting the CNS during glutamatergic hyperexcitation events. They observed that in APP/PSEN1 mice, the decrease in ascorbate levels was directly related to an increase in seizure episodes, cognitive impairment, and death rates (24). MOGAD paediatric patients may experience cognitive impairment and convulsions (25, 26). It has been shown that approximately 23% of paediatric MOGAD cases present seizures (27). Thus, reduced ascorbate and elevated glutamate levels in MOGAD may increase neuronal vulnerability to hyperexcitation states, potentially contributing to the higher frequency of seizures and cognitive symptoms reported in paediatric MOGAD. Nonetheless, the causal role of this relationship in this study remains speculative, and a targeted investigation should be conducted to deeply understand the role of these two metabolites in MOGAD.

### 4.2 Energy and lipid pathways

Energy metabolites like citric acid and mesaconic acid, one of its isomeric carboxylic acids, were reduced in our MOGAD patients compared to POMS patients, suggesting an impairment of the tricarboxylic acid (TCA) cycle and mitochondrial function.

Citrate is a central metabolite in cellular energy production, primarily involved in TCA. It is also essential for the biosynthesis of fatty acids, cholesterol, and lipids, which are critical components of the myelin sheath produced by oligodendrocytes. In MOGAD, oligodendrocytes are destroyed by the presence of antibodies; thus, low levels of citrate may simply reflect the unfavourable possibility of remyelination promoted by oligodendrocytes. Furthermore, acetyl-CoA, necessary for fatty acid synthesis and required for myelin formation, must be transported out of the mitochondria as a citrate derivative via the citrate shuttle. In this context, a low citrate level will hamper fatty acid biosynthesis and myelination (28). Abnormalities in energy metabolism and fatty acid biosynthesis have been reported in different diseases, including the neurodegenerative ones (29–32). Two different reviews reported that low citrate levels can be found in MS patients (12, 33). Accordingly, Kim et al. found low citrate levels in the CSF of MS compared to NMOSD patients, explaining this as a result of an increased activity of mitochondrial aconitase, which converts citrate to isocitrate (34). Surprisingly, in our study, POMS patients showed citrate levels similar to those of the controls. The explanations for this discrepancy may be different. Firstly, Kim and colleagues compared a demyelinating disease as MS with NMOSD, where the aetiology is related to dysfunctional astrocytes and does not involve oligodendrocytes and myelination processes. Instead, in our study, we compared two diseases, MOGAD and POMS, where the demyelination process is actively occurring. Secondly, the papers mentioned were investigating adult patients, whereas our study focused on paediatric patients. After birth and during early childhood, there is an increase in oligodendrocytes that promote myelination. This process actively continues and persists during adolescence up to adulthood. However, as soon as the disease progresses, high neuron, oligodendrocyte, and Oligodendrocyte Progenitor Cell (OPC) depletion could be observed. Increased inflammation level and loss of these cells will determine a lack of remyelination (35, 36). It may be that, in our POMS patients, the high citrate levels found were derived from a remaining functional oligodendrocyte reservoir that tried to respond to the pathological conditions promoting neuroprotective and regenerative remyelination.

Levels of dicarboxylic acid and amino fatty acid (e.g., 11-undecanoic, suberic, azelaic, and ethylmalonic acids) were highly enriched in MOGAD compared to POMS. The demyelination and remyelination processes occurring in these two diseases may explain our results. Asadollahi et al. demonstrated both *in vitro* and *in vivo* that in the case of an energy crisis, oligodendroglial fatty acid (FA) metabolism can serve as an energy reserve to avoid neuronal degeneration. Thus, we can speculate that the detection of elevated dicarboxylic and fatty acid components in the CSF of MOGAD patients is the consequence of myelin breakdown (37). Differently, in POMS, mainly at the early stages, oligodendrocytes are still functional and can uptake fatty acids to build and repair the myelin sheath. In addition, according to Židó et al., we can also conclude that in our treatment-naïve POMS patients, the active remyelination process was ongoing, reducing CSF fatty acid levels (38, 39).

Focusing on lipid composition, a recent study has shown results consistent with what we have found. Shi et al. analysed the CSF of adult patients affected by MS, aiming to identify lipids able to differentiate MS from other neuroinflammatory conditions, such as NMOSD. They reported that phosphatidylcholine (PC), TG, CE, PE, and PE-P were significantly reduced in the CSF of MS cases with gadolinium lesions. In addition, they demonstrated that CSF CE (16:0) inversely correlated with Expanded Disability Status Scale scores and may be considered a possible biomarker of disease progression and therapeutic response (40). In our study, the CSF of MOGAD patients exhibited a gain in short PE-P species like PE P-18:0_22:6, DG (P-23:0), and cholesterol ester (CE 18:2), whereas POMS patients had high levels of long PE (40:2 and 40:5) and TG (18:1-20:1-18:2). TG regulates metabolic storage through lipogenesis and lipolysis processes, which are important for organ integrity. Recent evidence has indicated that increased levels of TG are related to the onset and progression of various autoimmune diseases [e.g., Systemic Lupus Erythematosus (SLE)], including autoimmune demyelinating diseases (41, 42). In MS patients, high levels of TG have been reported to worsen the pathological condition as well as to increase the chances of recurrence of MS (43). It was hypothesised that TG may promote inflammation at the vascular endothelium and that immune cells passing the blood–brain barrier will cause the CNS dysfunction (44). Contrarily, Nogueras et al. showed that MS patients are characterised by increased levels of diacylglycerols and a decrease in TG. They claimed that this derives from a possible defect of the diacylglycerol acyltransferase (DGAT) enzyme, responsible for the conversion of DGAT to TG (45). Our metabolomic analysis clearly demonstrated that MOGAD patients, not POMS patients, had higher TG content. This difference can arise from the control/disease group characterised and chosen for the metabolic comparison. In Nogueras et al., the controls were defined just as patients with lumbar puncture and not suggestive of MS (45). MOGAD and MS are often difficult to discriminate at onset; therefore, finding TG specifically expressed only in MS samples supports that these are two completely different diseases and adds novel insight into the MS pathological condition. The high CE levels we found in MOGAD compared to POMS further sustain this idea. Normally, cholesterol represents 40% of the myelin composition. In healthy adults, CE is 0.2% of total CNS cholesterol; in case of myelin damage, the cholesterol released undergoes esterification and is packed in lipid droplets caught by the phagocytic microglia (46). Berghoff et al. showed that during the acute phase, oligodendrocytes can rescue the microglia’s recycled cholesterol, promoting remyelination, whereas a direct oligodendroglial cholesterol synthesis occurs during the chronic phase (47). In MOGAD patients, oligodendrocytes are destroyed by the antibodies, cholesterol cannot be recycled, and remyelination cannot actively occur, leading to an increase of cholesterol ester in the CSF. Different to our POMS patients who were in the initial phase of the disease, myelination can still happen, and cholesterol ester can be hydrolysed and used to reconstruct myelin (48).

All our findings align with a model in which antibody-mediated oligodendrocyte injury in MOGAD limits remyelination potential and disrupts lipid homeostasis. In contrast, the preservation of citrate levels in POMS may reflect residual oligodendrocyte function and partial regenerative capacity during early disease stages. Lipidomic profiling further supported divergent myelin repair capacities between MOGAD and POMS. Elevated cholesterol esters and short phosphatidylethanolamine plasmalogens in MOGAD likely reflect myelin breakdown and impaired cholesterol recycling by damaged oligodendrocytes. Conversely, higher triacylglycerol and long-chain phosphatidylethanolamine species in POMS are consistent with active lipid turnover associated with remyelination and neuroinflammatory repair processes. Taken together, these data indicate that MOGAD is primarily characterised by antibody-driven oligodendrocyte destruction and limited remyelination, whereas POMS retains partial regenerative potential.

### 4.3 Oxidative stress and neuroprotection

Our analysis has highlighted that oxidative stress modulation and neuroprotection seem to have a role in paediatric demyelinating diseases. Indeed, our MOGAD patients showed reduced levels of *N^8^*-acetylspermidine, ascorbic acid, and theobromine compared to POMS and control patients.

*N*^8^-Acetylspermidine is produced by the acetylation of spermidine. Polyamines are ubiquitous small polycations that ionically bind to various negatively charged molecules and have many functions, mostly linked to cell growth, survival, and proliferation. They increase the lifespan of different species and improve neural functions via the enhancement of autophagy in mice (49). Spermidine inhibits the synthesis of pro-inflammatory cytokines and promotes the autophagy of damaged cells. Our findings could be explained as a defensive reaction of the organism against autoimmune inflammation mediated by spermidine, leading to a significantly decreased acetylation to *N*^8^-acetylspermidine, which has neurotoxic properties. However, the relationship between spermidine and autoimmune inflammation has not yet been fully clarified (50–52). Another possible explanation could be linked to polyamines’ role in improving learning and memory. Indeed, their dysregulation has been linked to various diseases such as cancer, Snyder–Robinson syndrome, Parkinson’s disease, multiple system atrophy, and Alzheimer’s disease (AD). In AD, it has been demonstrated that polyamine dysregulation is dysfunctional and that acetylated polyamine products promote protein fibrillisation and oligomerisation, exacerbating neuronal damage (53). MOGAD is characterised by acute, monophasic, or relapsing inflammatory attacks, having autoantibodies specifically targeting myelin oligodendrocyte glycoprotein. Patients highly benefit from the prompt removal of these autoantibodies with immunosuppressive therapies. Differently, due to its chronic inflammatory nature, MS is characterised by progressive axon and myelin damage, involving long-term degenerative processes where macrophages play a key role. Thus, increased levels of polyamines could simply be the response of the body to contain this damage. Indeed, Yang et al. demonstrated that spermidine administration in an experimental autoimmune encephalomyelitis model inhibits macrophages’ pro-inflammatory activity and does not directly target pathogenic myelin-specific T cells (50).

Theobromine, a well-known caffeine metabolite, has a neuroprotective role too. Its effects have already been described in different diseases such as traumatic brain injury, Parkinson’s disease, Alzheimer’s disease, and MS (54–56). In our analysis, theobromine was low in MOGAD and high in POMS. The use of caffeine in children is not indicated; however, children can be excellent chocolate consumers. Theobromine is also known to be the principal alkaloid of the cacao bean (*Theobroma cacao*), explaining why we were able to detect this metabolite in our cohort of paediatric patients (57). The high level we detected in the CSF of POMS patients may just reflect the protective role of this substance against neurodegeneration processes, as already observed in other neurodegenerative diseases.

Ascorbic acid or vitamin C is a fundamental brain antioxidant and is involved in the synthesis of collagen, an important component of the myelin sheath (58). Generally, MS patients had a lower level compared to healthy subjects; however, the role of vitamin C in MS is still under debate. Some authors did not find any differences between disease and control patients in daily vitamin C intake (59). Conversely, others claimed that the decreased levels of vitamin C observed in MS patients may result from either increased lipid peroxidation or an elevated oxidative stress (60). Our cohorts were paediatric patients at the onset of the disease, where an initial antioxidative protective resistance could be observed. During the progress of the disease, this effect will vanish due to the overriding impact of neurodegeneration (46).

Our data seem to indicate a potential oxidative resilience in POMS due to the high theobromine levels, whereas ascorbate depletion in MOGAD may indicate increased oxidative load or impaired recycling under acute inflammatory stress. However, further studies integrating redox markers and mitochondrial function may help in elucidating how oxidative balance influences demyelination severity and recovery potential in these two diseases.

### 4.4 Developmental characteristic: paediatric ADS versus adult MS

Our findings highlight both shared and developmentally distinct biochemical features between paediatric demyelinating diseases and adult-onset multiple sclerosis (MS).

Smusz et al. have shown that adult MS typically shows reduced TCA intermediates such as citrate and succinate, reflecting mitochondrial dysfunction and chronic energy insufficiency (61). In our paediatric data, similar reductions in citric acid and mesaconic acid were observed only in MOGAD patients, suggesting comparable energy pathway disruption. Instead, POMS patients showed normal citrate levels, possibly reflecting developmental resilience and residual mitochondrial capacity during ongoing myelination.

Lipid metabolism also showed distinct age-related patterns. Adult MS is characterised by chronic phospholipid loss and ceramide accumulation, reflecting sustained lipid oxidation and impaired membrane renewal (61). In contrast, in our study, paediatric MOGAD patients showed elevated cholesterol esters, reflecting acute antibody-mediated myelin damage and defective cholesterol recycling, whereas POMS patients displayed increased triacylglycerols and long-chain phosphatidylethanolamines, consistent with active lipid remodelling and early repair, suggesting partial regenerative potential during development.

Disruptions in amino acid metabolism and lipid homeostasis were shown by Židó et al., even at MS onset (62). They demonstrated significant decreases in arginine and histidine levels and an increase in palmitic acid in treatment-naïve adult patients at the first MS attack (62). These findings complement our paediatric observations. Histidine depletion and altered derivatives were evident in both POMS and MOGAD, suggesting a conserved involvement of histidine pathways across developmental stages; however, their interpretation may differ. In adults, histidine consumption likely reflects chronic neuroinflammatory activation and histamine synthesis, whereas in paediatric patients, the increased *N*,*N*-dimethyl-l-histidine detected in MOGAD may represent an adaptive, neuroprotective response. Similarly, the increase in palmitic acid reported by Židó et al. aligns with our lipidomic evidence of disrupted fatty acid metabolism, particularly in MOGAD, where elevated cholesterol esters suggest acute myelin catabolism.

This comparison of adults and paediatric datasets supports a possible developmental continuum of metabolic vulnerability. Early-onset MOGAD and POMS are marked by acute, antibody- and inflammation-driven metabolic disruption with partial compensatory potential, whereas adult MS exhibits entrenched mitochondrial dysfunction and lipid depletion linked to cumulative demyelination. Recognising these stage-specific metabolic signatures may be crucial for designing age-appropriate therapeutic strategies and for identifying biomarkers that distinguish transient inflammatory damage from chronic neurodegenerative change.

### 4.5 Limitations and future directions

The nature, the phase, and the age of our cohort may explain our results; however, the small patient sample size is an obvious limitation of the study, dictated by the relative rarity of these conditions, especially since only immunotherapy-naïve patients were selected.

Group comparisons were not adjusted for age, sex, pubertal status, Body Mass Index (BMI), or CSF biochemistry; therefore, we acknowledge that some of the observed differences may partially reflect cohort heterogeneity. Diet and postprandial changes in plasma metabolites may also affect plasma/CSF exchange and CSF metabolite concentrations. To minimise this source of variability, all children in our cohort underwent lumbar puncture in the morning, after fasting for at least 6 hours. Indeed, morning collection was chosen to minimise circadian variability, and fasting was ensured, per clinical protocol, to reduce dietary effects on CSF composition. Moreover, the presence of two oligoclonal band (OCB)-positive MOGAD patients introduced biological heterogeneity that may have influenced the group’s metabolomic profile, as OCB positivity has been associated with a more inflammatory clinical phenotype in paediatric MOGAD (63).

In addition, our study focused on metabolomic analysis only in the CSF of our paediatric patients. CSF reflects the actual state of the CNS during inflammatory processes, it is fundamental to understand the pathology of MOGAD and POMS, and it is also considered a “gold standard matrix” in the diagnosis of ADSs. However, CSF is a biological specimen obtained by a procedure that is still highly invasive, especially in children. Further future analysis should be performed on serum samples to validate the results we found in the CSF to find novel serum biomarkers and avoid lumbar puncture in children.

### 4.6 Conclusions

In conclusion, this metabolomic analysis provides preliminary evidence of metabolites that differentiate paediatric MOGAD from POMS at disease onset in treatment-naïve patients. To our knowledge, this is the first study to directly compare CSF metabolomic profiles between these two conditions. Our data show that glutamate and ascorbate levels inversely correlate and may be key metabolites in the development of seizures and intellectual disability observed in MOGAD. Moreover, low cholesterol levels in MOGAD and high TG content in POMS could be distinctive traits of these two diseases, mirroring the absence or presence of an active myelinating process and inflammation. It should be acknowledged that the metabolomic signatures observed in the MOGAD group should be interpreted with caution, as they may be influenced in part by the presence of OCB-positive patients. For these reasons, validation in larger and more homogeneous cohorts will be essential to confirm these preliminary observations and further clarify their biological relevance. Future studies with sufficient statistical power to stratify MOGAD patients by OCB status will be critical to determine the specific impact of this immunological subset on CSF metabolomic profile.

## Data Availability

The raw data supporting the conclusions of this article will be made available by the authors, without undue reservation.
