# Supplementary material for: Cerebrospinal fluid metabolomic signatures in paediatric MOGAD and POMS
Source: Front Immunol. 2026 Jan 2;16:1650785. doi: 10.3389/fimmu.2025.1650785 (PMC12808355; doi:10.3389/fimmu.2025.1650785)
Supplement: Supplementary file 1 [file DataSheet1.docx]

**Supplementary Materials**

**Sample characterization**

**Indirect immunohistochemistry and immunofluorescence.**

For intracellular neuronal antibodies, perfused rat cerebellum sections were incubated with 10% goat serum for 30 minutes at room temperature. The sections were then incubated with patients’ or control CSF (1:2) for 3 hours at 37°C. Then the sections were first incubated with biotinylated goat antihuman IgG (Vector lab) for 30 minutes and then with avidin-biotin peroxidase for 30 minutes at RT. For antigen surface antibodies, rat brains were fixed for 1h in 4% paraformaldehyde at 4°C and cryoprotected with 40% sucrose for 48h, then embedded in freezing medium, snap frozen in isopentane chilled with liquid nitrogen. Seven µm brain cryosections were incubated with 0.3% hydrogen peroxide for 15 minutes and then incubated with 5% goat serum for 1h. The sections were then incubated with patients’ or control CSF (1:2) overnight at 4°C. The day after, the sections were first incubated with biotinylated goat antihuman IgG (Vector lab) for 2h and then with avidin-biotin peroxidase for 1h at RT. For both intracellular and surface antibodies, the final reactivity was developed with diaminobenzidine (Dako).

**Metabolomic and Lipidomic analysis**

**Sample preparation for UHPLC-M**

UHPLC-MS analysis

Ultrahigh performance liquid chromatography (UHPLC) was carried out using an Ultimate 3000 system from Thermo Fisher Scientific coupled to a classic QExactive by a heated ESI source (HESI). An Accucore 150 Amide HILIC UPLC column (100 × 2.1 mm, 2.6 μm, Thermo Fisher Scientific) was used at 35°C for the separation of polar metabolites. Mobile phases consisted of acetonitrile and H_2_O (95:5, v/v) with 10 mM of ammonium formate and 0.1% of formic acid (Phase A), and acetonitrile and H_2_O (50:50, v/v) with 10 mM of ammonium formate and 0.1% of formic acid (Phase B). The flow rate was 450 µl/min, and the injection sample volume was 2 µl in positive ionization mode and 4 µl in negative mode. All samples were maintained at 15°C during the analysis.

A reverse phase column (C18 Hypersil gold, 150 × 2.1 mm, 1.9 μm, Thermo Fisher Scientific), a Column guard—Hypersil Gold 3 μm, and a Column guard holder were used at 55°C for non-polar metabolites separation. Mobile phases consisted of acetonitrile and water (60:40, v/v) with 10 mM of ammonium formate and 0.1% of formic acid (Phase A), and acetonitrile and 2-propanol (10:90, v/v) with 10 mM of ammonium formate and 0.1% of formic acid (Phase B). The flow rate was 400 µl/min, and the sample injection volume was 5 µl in positive ionization mode (ESI+) and 6 µl for negative ionization mode (ESI-). All samples were maintained at 10°C during the analysis.

Table 1 shows the gradient used for polar and non-polar metabolites profiling by UHPLC-MS.

Supplementary Table 1

| Polar Metabolites | | | Non-polar Metabolites | | |
| --- | --- | --- | --- | --- | --- |
| Time | Phase A | Phase B | Time | Phase A | Phase B |
| 0 | 99 | 1 | 0 | 70 | 30 |
| 1 | 99 | 1 | 0.5 | 70 | 30 |
| 2 | 85 | 15 | 2 | 57 | 43 |
| 12 | 5 | 95 | 2.1 | 45 | 55 |
| 14 | 5 | 95 | 12 | 35 | 65 |
| 14.5 | 99 | 1 | 18 | 32 | 85 |
| 20 | 99 | 1 | 20 | 0 | 100 |
|  |  |  | 26 | 0 | 100 |
|  |  |  | 26.1 | 70 | 30 |
|  |  |  | 31 | 70 | 30 |

Data were acquired in positive and negative ionization modes separately with a resolution of 70000 (FWHM at m/z 200).

Ion source parameters:

For polar metabolites: sheath gas = 45 arbitrary units (AU), aux gas = 12 AU, spray voltage = 3.2 kV, capillary temperature = 300°C (ESI+)/270°C (ESI-), probe heater temperature = 320°C (ESI+)/290°C (ESI-). Data-dependent acquisition was applied to the pool samples over three mass ranges (70-160 m/z, 161-250 m/z, 251-1050 m/z) using the following settings: resolution = 17500; isolation width = 1.5 m/z; stepped normalized collision energies = 30, 50, and 90% (ESI+)/30, 60 and 100% (ESI-).

For non-polar metabolites: sheath gas = 48 AU, aux gas = 15 AU, spray voltage = 3.2 kV (ESI+)/ 2.7 kV (ESI-), capillary temperature = 320°C (ESI+)/ 350°C (ESI-), probe heater temperature = 400°C (ESI+)/320°C (ESI-). Data-dependent acquisition was applied to the pool samples over three mass ranges (250-500 m/z, 501-700 m/z, 701-1200 m/z) using the following settings: resolution = 17500; isolation width = 1.5 m/z; stepped normalized collision energies = 10, 20, and 30% (ESI+)/24, 24 and 28% (ESI-).

Supplementary Table 2

| Compound Name | Predicted Formula | Detected  m/z | Fragmentation pattern | Ion description | Area  MOGAD | Area  POMS | Area Control | Identification level |
| --- | --- | --- | --- | --- | --- | --- | --- | --- |
| PE  (P-18:0_22:6) | C_45_H_78_NO_7_P | 776.55159 | 392.29138, 385.27347, 121.10075, 109.10135, 107.08531, 95.06987, 93.06987, 81.06980, 69.06985, 67.05426 | [M+H]+ | 4672620  (974388-8524526) | 1121161  (267715-1432292) | 66150  (59710-134647) | 2 |
| Pipercitine | C_23_H_43_NO | 349.33455 | 350.24219: 112.07565: 86.09644: 69.0699 | [M+H]+ | 333801  (73865-1488503) | 8070  (7322-10096) | 9378  (7148-21872) | 2 |
| 2,2'-(2,6-Pyridinediyl)bis(N,N,N',N'-tetramethyl-1,3-propanediamine) | C_19_H_37_N_5_ | 336.31197 | 366.31158, 155.15472 | [M+H]+ | 1429720  (410897-2543301) | 62876  (26861-118393) | 52924  (19468-81141) | 2 |
| (4E)-4-(Hexadecylimino)pentanoic acid | C_21_H_41_NO_2_ | 340.32110 | 340.32129, 95.08544, 88.07568, 71.0856, 57.0701 | [M+H]+ | 426979  (70259-3136951) | 20241  (13154-24942) | 21950  (18545-25103) | 2 |
| DG  (P-6:0_17:0) | C_26_H_50_O_4_ | 427.37822 | 157.12250, 121.10122, 111.11673, 97.10115, 95.08568, 93.06984, 81.06979, 79.05377, 71.08556, 69.07007 | [M+H]+ | 201087  (68647-853204) | 10469  (8579-12150) | 153587  (112874-201810) | 2 |
| CE 18:2 | C_45_H_76_O_2_ | 671.57364 | 671.61639, 620.09149,303.22971 | [M+Na]+ | 1571376  (115277-4133532) | 64756  (57749-85290) | 57907  (56517-90826) | 2 |
| 10-Hydroxydecanoi c acid | C_10_H_20_O_3_ | 187.13414 | 125.09721, 97.06625 | [M-H]- | 385571  (163369-504750) | 121642  (111559-163890) | 108218  (95492-111502) | 2 |
| N_N-Dimethyl-L-histidine | C_8_H_13_N_3_O_2_ | 184.10813 | 184.10846, 166.09814, 72.04467, 71.04933 | [M+H]+ | 10074027  (2654933-27265881) | 1154040  (678263-1409427) | 1121714  (838961-1890786) | 2 |
| N-Methyl-N-(1-phenylethyl)-1-hexadecanamine | C_25_H_45_N | 360.36247 | 360.36230, 268.29974, 91.05425, 58.0654 | [M+H]+ | 49452141  (42730989-53782511) | 15080937  (11779445-55032495) | 38062520  (32264669-57349443) | 2 |
| 11-Aminoundecanoic acid | C_11_H_23_NO_2_ | 202.18022 | 202.18048, 144.10223, 123.11702, 98.0965, 84.08092, 67.0545, 57.0702 | [M+H]+ | 126334853  (42642839-172421171) | 14710889  (10744271-76111832) | 20560470  (11107624-32297378) | 2 |
| D-Glutamate | C_5_H_9_NO_4_ | 148.06052 | 147.07630, 130.04979, 84.04434 | [M+H]+ | 1716081  (599458-2736325) | 349128  (299953-574807) | 389261  (290515-619060) | 2 |
| D-Glutamate | C_5_H_9_NO_4_ | 146.04604 | 102.05613, 59.01327 | [M-H]- | 2539881  (803820-3660023) | 531004  (403100-755406) | 492098  (425567-885792) | 2 |
| Capryloylglycine | C_10_H_19_NO_3_ | 200.12947 |  | [M-H]- | 370979  (215882-1006074) | 174892  (134614-257564) | 168370  (129298-179770) | 3 |
| Isoleucine | C_6_H_13_NO_2_ | 132.10199 | 132.10225, 74.06018, 56.04967 | [M+H]+ | 2595363  (520358-5917289) | 354979  (169045-422700) | 356967  (269683-433458) | 2 |
| Ethylmalonic acid | C_5_H_8_O_4_ | 131.03505 | 87.04501 | [M-H]- | 1355563  (624601-2024478) | 357084  (304133-800098) | 404752  (330388-461391) | 2 |
| Nicotinamide | C_6_H_13_N_2_O | 123.05534 | 123.05542, 80.04963, 79.04172 | [M+H]+ | 1713020  (760295-2589380) | 705718  (498016-1102408) | 566736  (317985-700151) | 2 |
| Suberic acid | C_8_H_14_O_4_ | 173.08208 | 173.08321, 129.09227, 111.08177, 83.05032, 57.03427 | [M-H]- | 6237414  (3190563-10758360) | 2299542  (1959267-3487748) | 2353875  (1873029-2859072) | 2 |
| Azelaic acid | C_9_H_16_O_4_ | 187.09781 | 187.09700, 125.09721, 97.06625 | [M-H]- | 10888228  (5778962-17827699) | 4030795  (3764009-7213502) | 4013338  (3352408-4561682) | 2 |
| Capryloylglycine | C_12_H_23_NO_3_ | 230.17516 | 212.16492, 167.14297, 166.15926, 135.1169, 107.08588, 93.0700, 83.08574, 69.07008, 55.0546 | [M+H]+ | 665056  (192003-1606420) | 140933 (  121651-166066) | 102732  (87127-146863) | 2 |
| Piperidine | C_5_H_11_N | 86.09643 | 86.09654, 69.07007, 67.05447 | [M+H]+ | 29573434  (20378647-80460215) | 13567552  (12481677-13853043) | 12174430  (10847421-13682217) | 2 |
| N-Acetyl-1-aspartylglutamic acid | C_11_H_16_N_2_O_8_ | 305.09786 | 158.04559, 148.06079, 130.05014, 102.05507, 88.03946, 70.02897 | [M+H]+ | 3897894  (2467688-5737530) | 1891901  (1521375-2998872) | 3515373  (2393977-6516240) | 2 |
| PE 40:5 | C_45_H_80_NO_8_P | 794.56971 | 794.56360, 653.55072 | [M+H]+ | 421544  (109335-694079) | 1112161  (267715-1432292) | 64003  (62245-151569) | 2 |
| PE 40:4 | C_45_H_82_NO_8_P | 796.58479 | 796.58624, 655.56622 | [M+H]+ | 365614  (78871-588402) | 1184888  (963145-1916583) | 93992  (82766-97259) | 2 |
| N8-Acetylspermidine | C_9_H_21_N_3_O | 188.17587 | 188.17615, 171.14948, 112.11214, 100.07586, 84.0808, 72.0896 | [M+H]+ | 3203570  (1892491-13577958) | 12287063  (11455845-16633269) | 13065733  (11165514-14884164) | 2 |
| Mesaconic acid | C_5_H_6_O_4_ | 129.01948 | 96.32028, 67.01857 | [M-H]- | 686954  (490529-24444925) | 26081862  (5308997-44800949) | 13707390  (3796060-20988985) | 2 |
| TG  (18:1_20:1_18:2) | C_59_H_106_O_6_ | 928.83356 | 930.84890, 629.55170, 603.53503 | [M+NH4]+ | 1025111  (265046-2500137) | 5733099  (2347392-13235005) | 809589  (524369-2283886) | 2 |
| Citric acid | C_6_H_8_O_7_ | 191.01996 | 71.01373, 67.01872 | [M-H]- | 1052309  (600608-9203652) | 8236582  (4774537-13140564) | 266603  (95492-111502) | 2 |
| 3-methylistidine | C_7_H_11_N_3_O_2_ | 170.09250 | 170.09265, 153.06564, 126.10288, 125.07111, 109.07629, 96.06820, 95.06051 | [M+H]+ | 268739  (113396-1794914) | 6531245  (3520595-8358121) | 4616007  (202504-6166050) | 2 |
| Theobromine | C7H8N4O2 | 181.07202 | 181.07239, 163.06139, 138.06628, 137.08218, 110.07127, 108.05563, 69.04472, 67.02908 | [M+H]+ | 428995  (123760-2402359) | 4594983  (1206491-19703953) | 4418783  (1871125-14211344) | 2 |
| Ascorbic acid | C_6_H_8_O_6_ | 175.02498 |  | [M-H]- | 26138  (18308-436114) | 307077  (57596-511882) | 266603  (150391-416093) | 3 |

**Supplementary Figures**

**Suppl. Fig. 1. Tissue-based assay analysis.**

Representative images of indirect immunofluorescence assay on cerebellum section of: a nuclear positive control sample (A), a Purkinje cytoplasmic positive control sample (B), a negative control sample (C), a MOGAD patient sample (D), a POMS sample (E), and IIH patient sample (F). All the images were acquired with the 20X magnification objective.


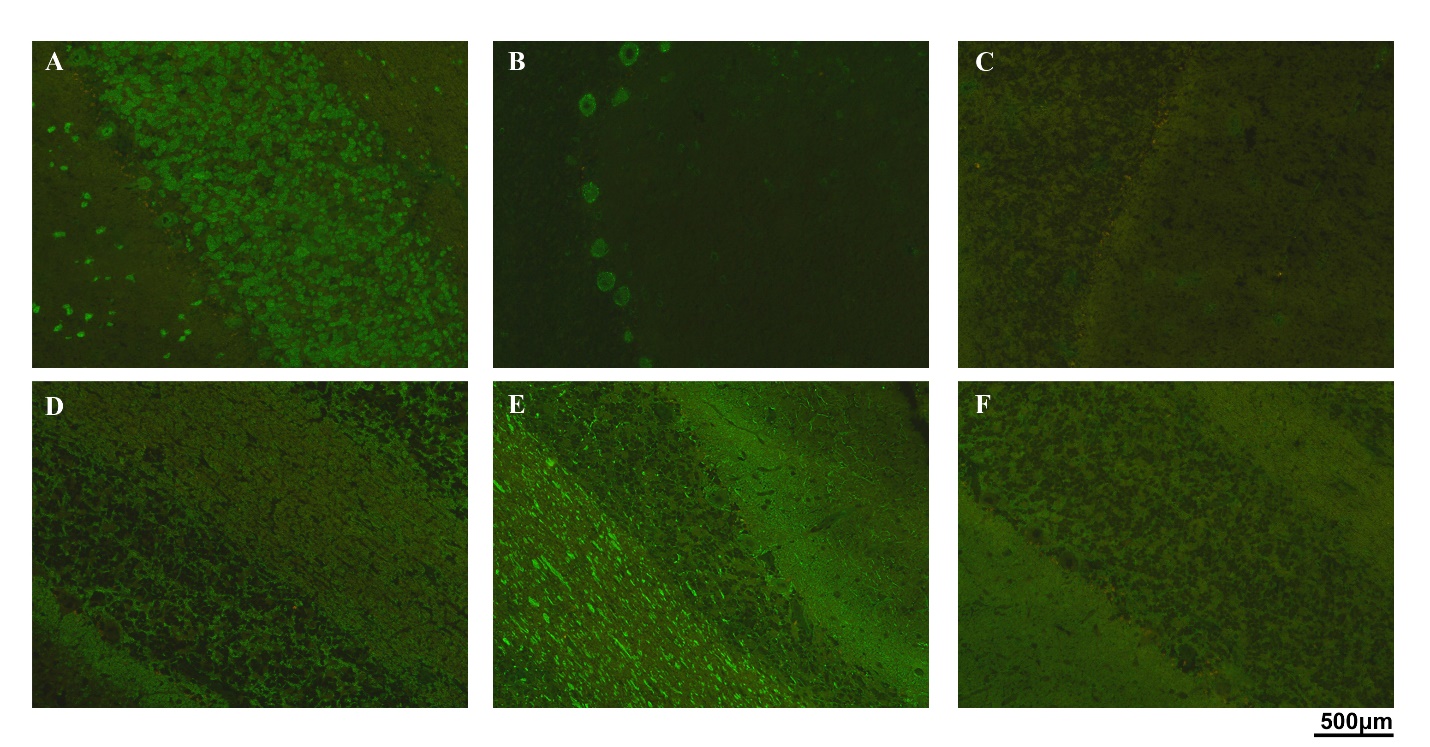


**Suppl. Fig. 2. Additional immunological analysis: line blot and cell-based assay.**

Solid phase test and cell-based assay analysis to check for the presence of other antineuronal antibodies. **a.** Representative image of a sample resulted negative for intracellular neuronal antibodies using line blot. **b.** Representative images of a sample positive for NMDAR antibodies, analysed using a biochip containing six different neuronal antibodies target (NMDAR, CASPR2, LGI1; GABA_b_, AMPAR1/2, DPPX). All the images were acquired with the 20X magnification objective.


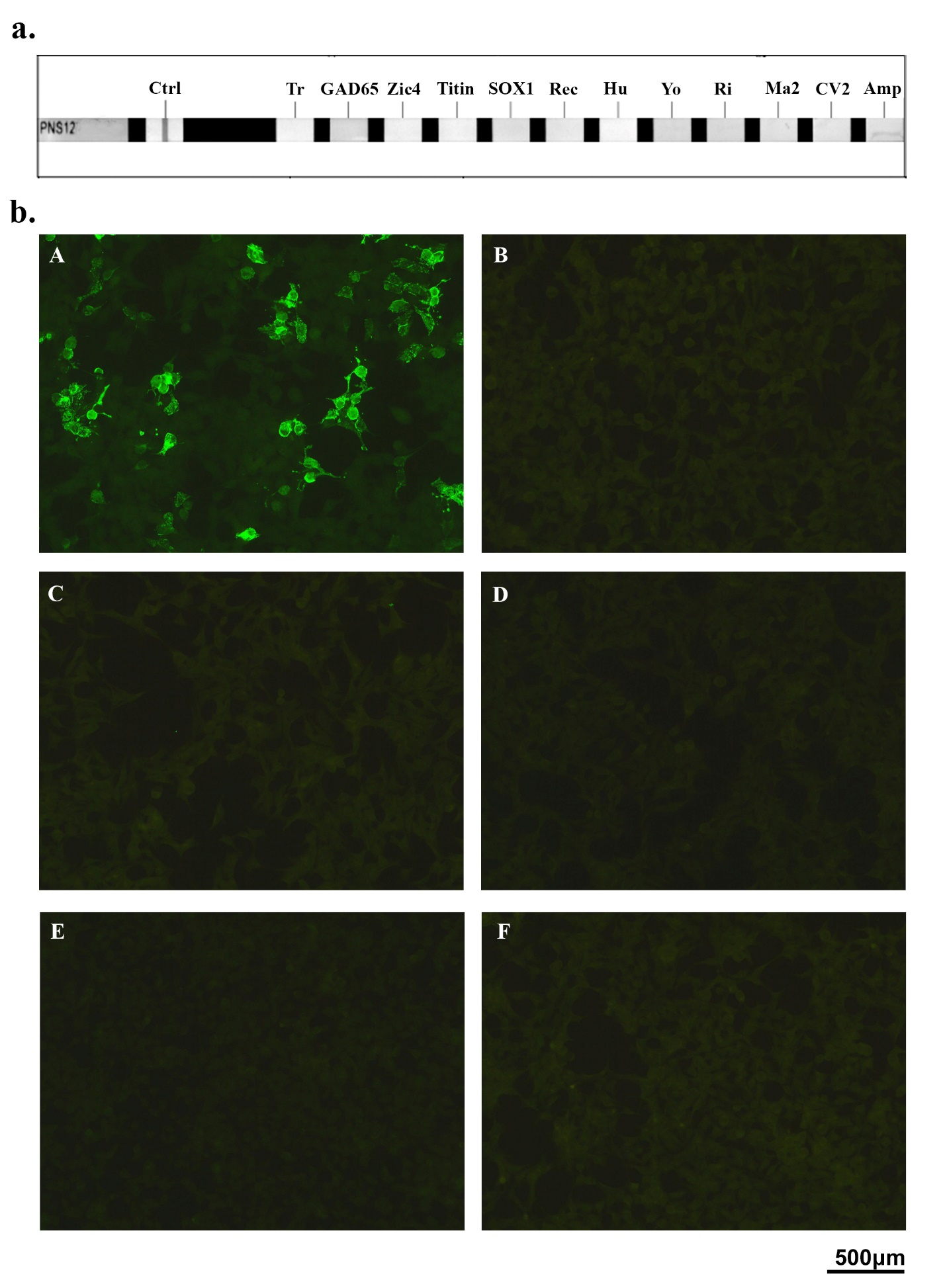


**Suppl. Fig. 3. sPLS-DA plot**

Polar Metabolites, Positive Ionisation


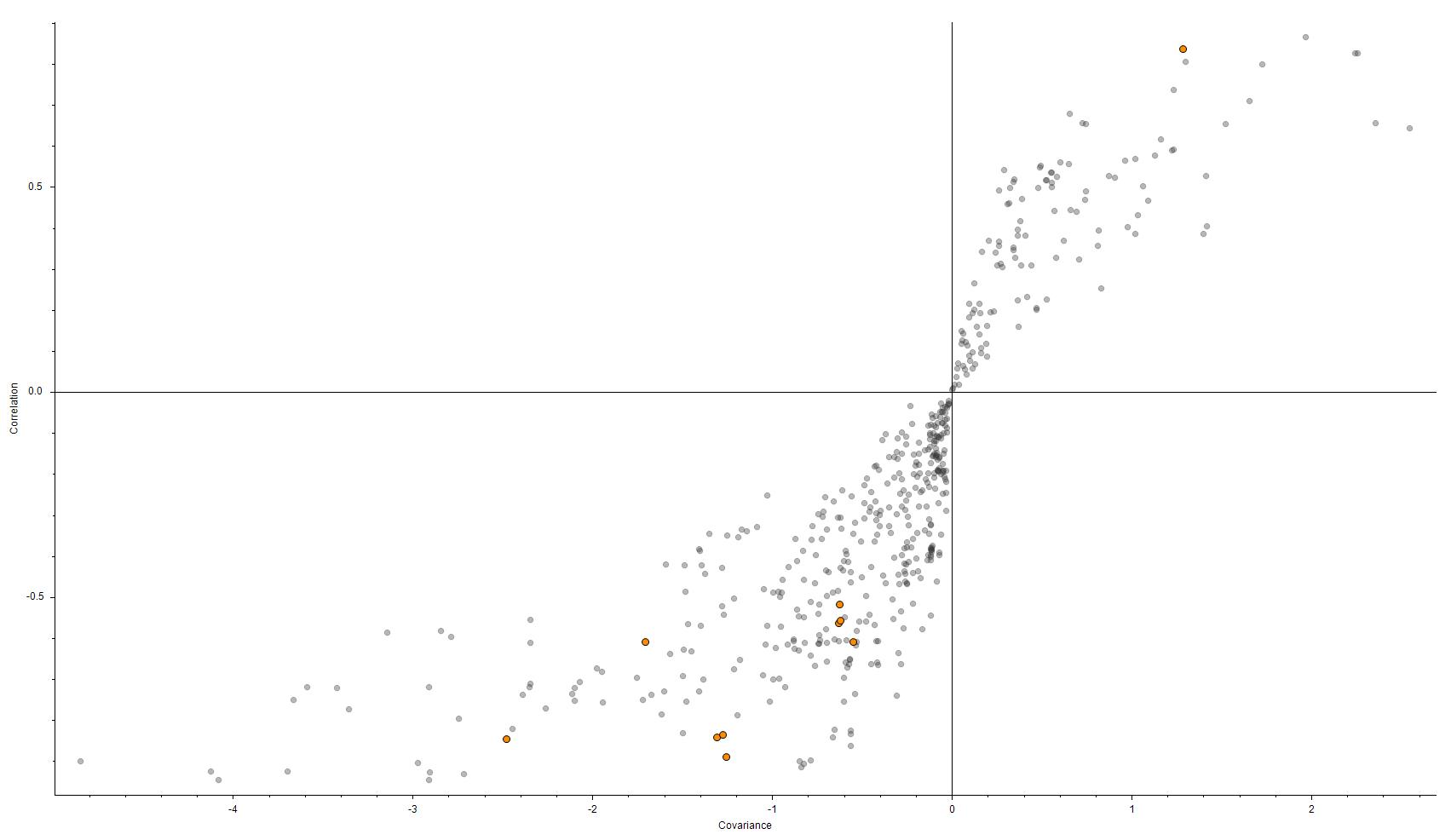


Polar Metabolites, Negative Ionisation


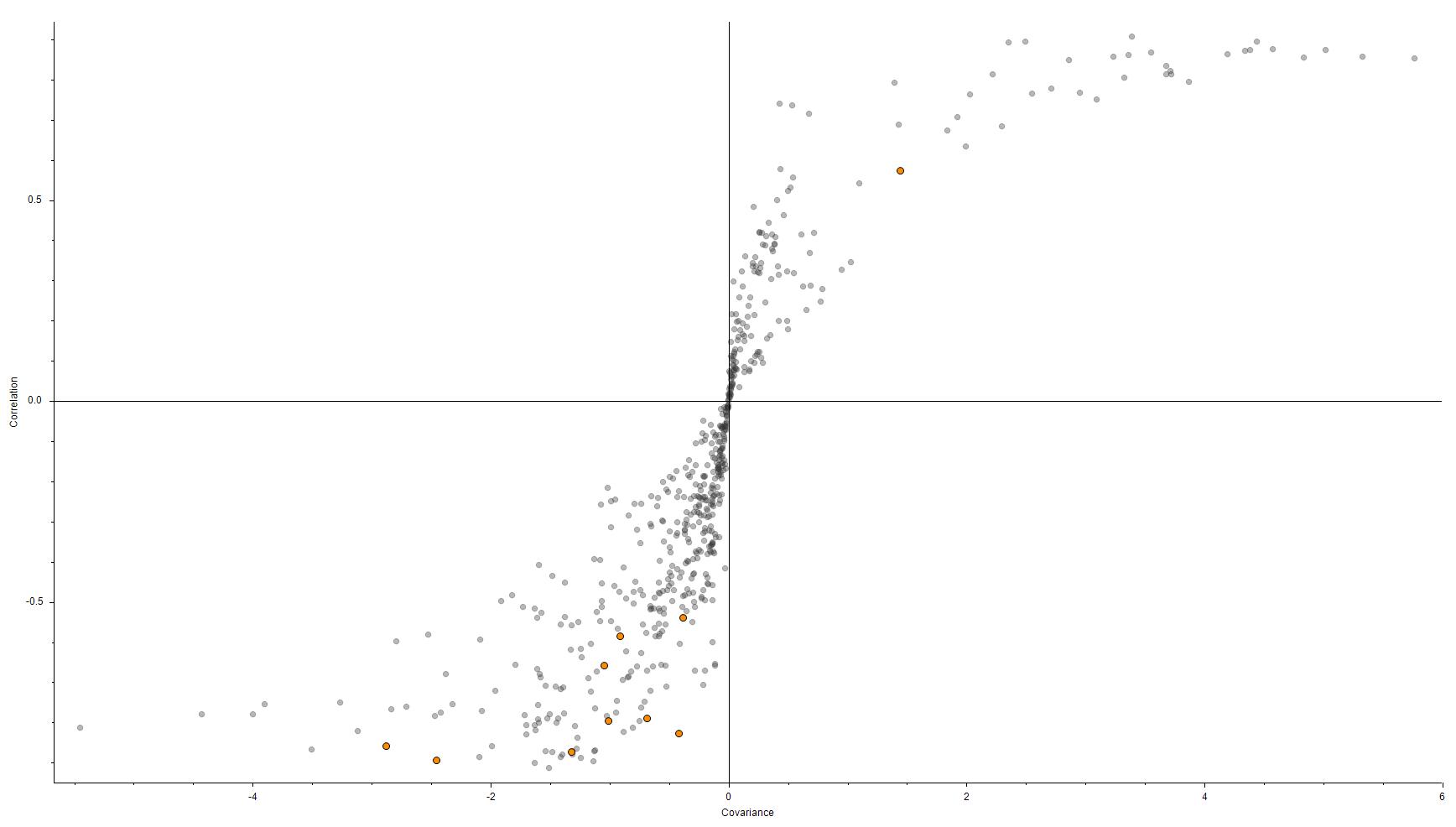


Non Polar Metabolites, Positive Ionisation


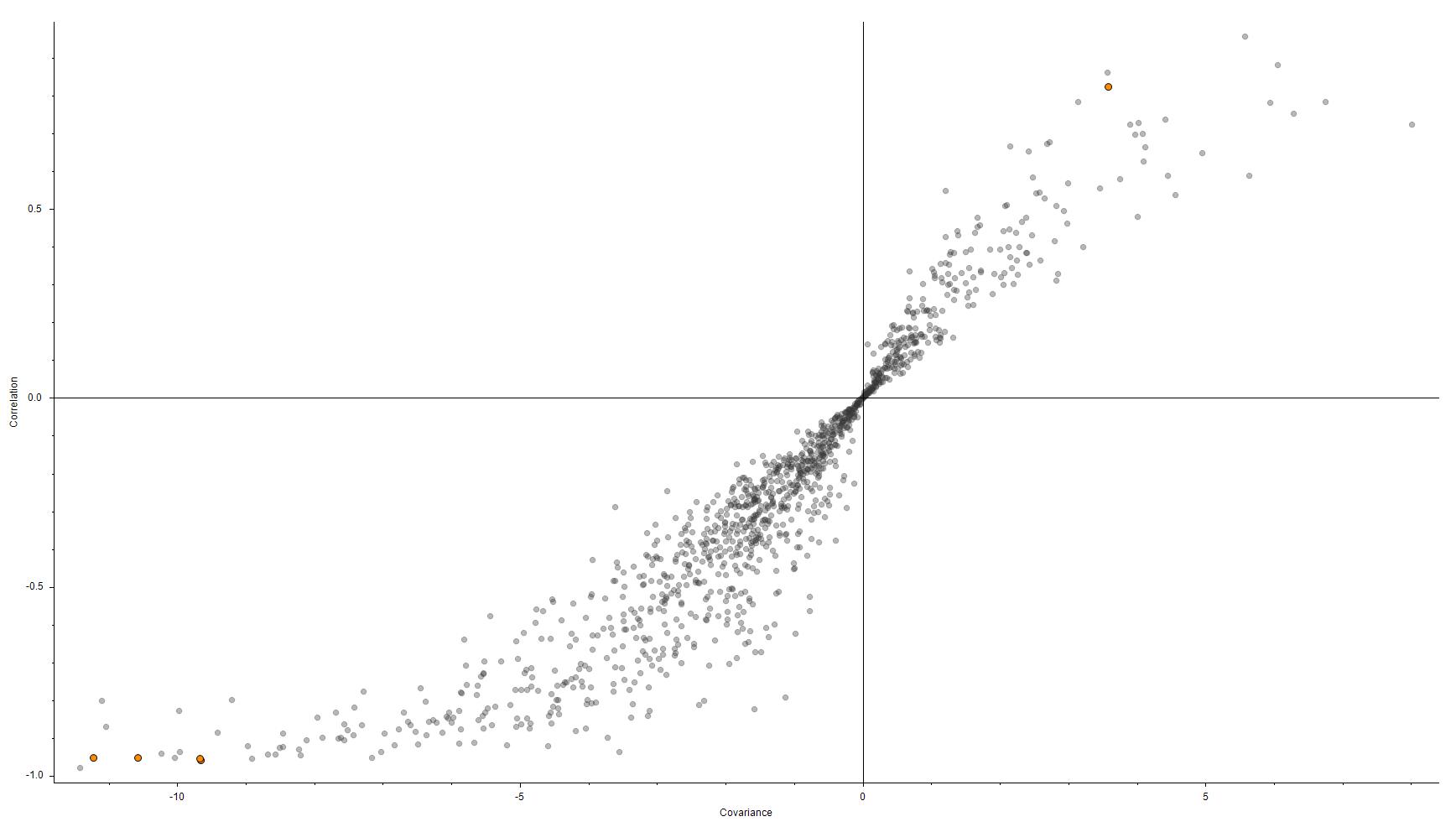


Non Polar Metabolites, Negative Ionisation


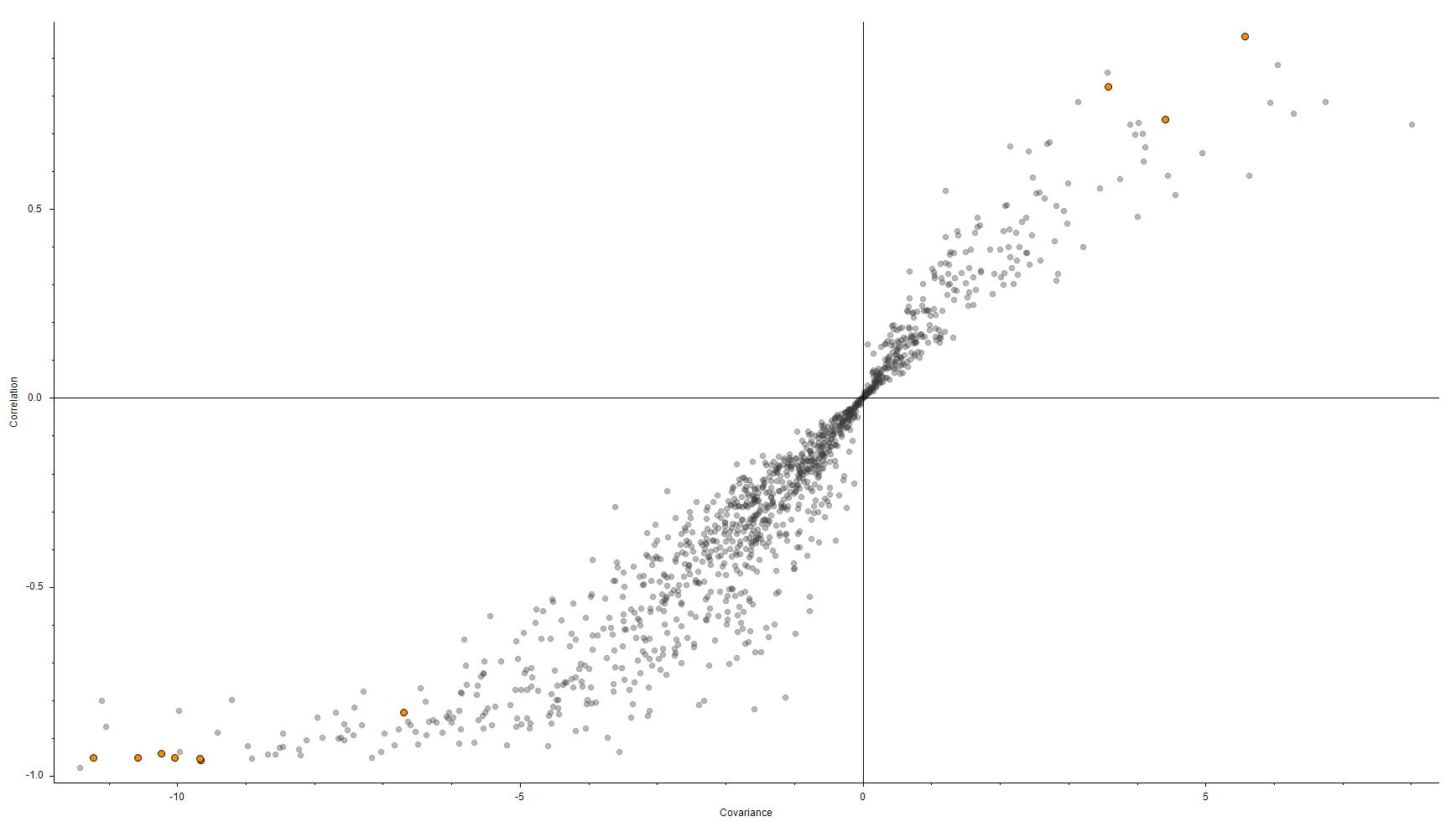


PLS-DA of Polar and non-polar metabolites for positive and negative ionization with normalized area. The orange circles represent the discriminating compounds.
